# ﻿Description of the complete rDNA repeat unit structure of *Coturnixjaponica* Temminck et Schlegel, 1849 (Aves)

**DOI:** 10.3897/compcytogen.18.127373

**Published:** 2024-09-25

**Authors:** Alina Zhukova, Gennadii Zakharov, Olga Pavlova, Alsu Saifitdinova

**Affiliations:** 1 Herzen State Pedagogical University of Russia, Saint Petersburg, Russia Herzen State Pedagogical University of Russia Saint Petersburg Russia; 2 Pavlov Institute of Physiology, Russian Academy of Sciences, Saint Petersburg, Russia Pavlov Institute of Physiology, Russian Academy of Sciences Saint Petersburg Russia; 3 EPAM Systems Inc., Saint Petersburg, Russia EPAM Systems Inc. Saint Petersburg Russia; 4 International Centre for Reproductive Medicine, Saint Petersburg, Russia International Centre for Reproductive Medicine Saint Petersburg Russia; 5 Beagle Ltd., Saint Petersburg, Russia Beagle Ltd. Saint Petersburg Russia; 6 Saint Petersburg State University, Saint Petersburg, Russia Saint Petersburg State University Saint Petersburg Russia

**Keywords:** Bendable DNA, nanopore sequencing, Japanese quail, nucleolar organizer, RNA polymerase I promoter

## Abstract

Ribosomal RNA (18S, 5.8S, 28S) gene clusters in genomes form regions that consist of multiple tandem repeats. They are located on a single or several pairs of chromosomes and play an important role in the formation of the nucleolus responsible for the assembly of ribosome subunits. The rRNA gene cluster sequences are widely used for taxonomic studies, however at present, complete information on the avian rDNA repeat unit structure including intergenic spacer sequence is available only for the chicken (*Gallusgallusdomesticus* Linnaeus, 1758). The GC enrichment and high-order repeats peculiarities within the intergenic spacer described for the chicken rDNA cluster may be responsible for these failures. The karyotype of the Japanese quail (*Coturnixjaponica* Temminck et Schlegel, 1849) deserves close attention because, unlike most birds, it has three pairs of nucleolar organizer bearing chromosomes, two of which are microchromosomes enriched in repeating elements and heterochromatin that carry translocated terminal nucleolar organizers. Here we assembled and annotated the complete Japanese quail ribosomal gene cluster sequence of 21166 base pairs (GenBank under the registration tag BankIt2509210 CoturnixOK523374). This is the second deciphered avian rDNA cluster after the chicken. Despite the revealed high similarity with the chicken corresponding sequence, it has a number of specific features, which include a slightly lower degree of GC content and the presence of bendable elements in the content of both the transcribed spacer I and the non-transcribed intergenic spacer.

## ﻿Introduction

**Figure 1.**
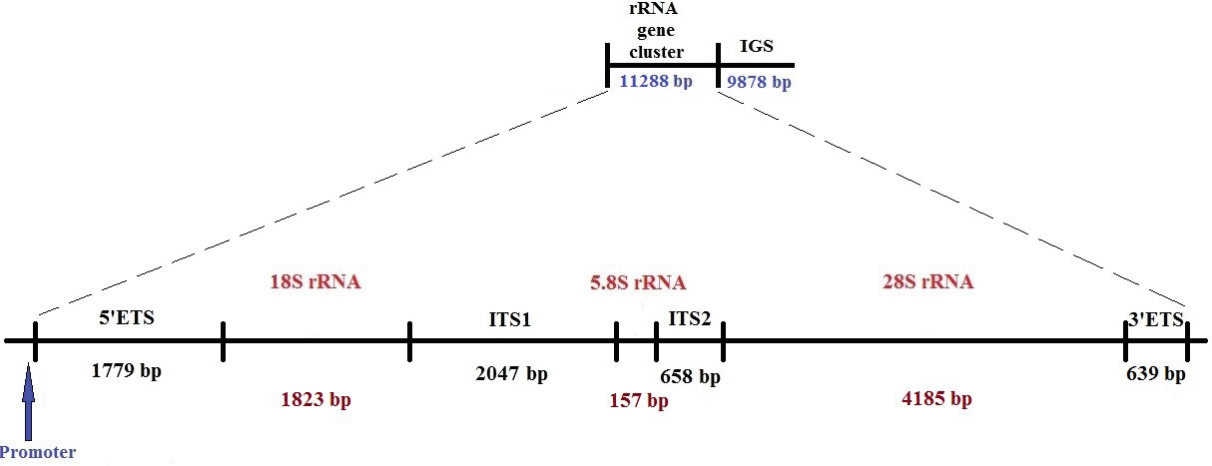
rRNA gene cluster of *C.japonica*.

Genomic elements responsible for conservative processes are of interest both from the point of view of studying the mechanisms of their implementation and for solving various molecular taxonomic problems. Protein synthesis or translation is one of the key and most ancient cellular processes. Translation takes place on ribosomes - complex particles of large and small subunits each consisting of two-thirds of ribosomal RNAs (rRNAs) and one-third of ribosomal proteins (Alberts et al. 2002). It is rRNA molecules, not proteins, that are responsible for the overall three-dimensional structure of the ribosome, the correct positioning of tRNA on the mRNA template, and enzymatic activity in creating peptide bonds (Ban et al. 2000; Alberts et al. 2002). In the animal cell there are four types of highly conservative rRNAs: 18S, a component of a small ribosomal subunit, and 5S, 5.8S, 28S forming a large ribosomal subunit. The 5S rRNA gene copies transcribed by RNA polymerase III typically make up a separate locus in the genome whereas the 18S, 5.8S, 28S rRNA genes are situated together in a cluster (Fig. 1), which is repeated many times forming loci of the ribosomal DNA (rDNA) generally referred to the nucleolus organizer regions. rDNA cluster the genes are separated by internal transcribed spacers (ITS1 and ITS2) and flanked by external transcribed spacers, 5’-ETS at the 5’-end the 18S rRNA gene and 3’-ETS at the 3’-end of the 28S rRNA gene. Transcription of rDNA clusters is operated by RNA polymerase I complex synthesizing a single precursor rRNA molecule (47S pre-rRNA), which is subsequently processed in three separate rRNAs. rDNA clusters are separated by an intergenic spacers (IGS), together they form rDNA repeated units (Alberts et al. 2002). The IGS comprises various inner repeats, and their deletion or amplification are the main causes of the IGS length variability, e.g. from ~3.3 kb in *Drosophilapseudoobscura* Frolova et Astaurov, 1929 (Mateos and Markow 2005) up to ~30 kb in human (Hori and Shimamoto 2021). The IGS contains rRNA promoter and terminator sites, regulatory elements, such as enhancer elements, which control pre-rRNA synthesis (Goodfellow and Zomerdijk 2013). Replication origins and replication fork barriers (RFBs) that prevent conflicts between the replication and transcription machineries are also located in the IGS (Brewer et al. 1992; Kobayashi et al. 1992; Akamatsu and Kobayashi 2015). Analysis of rDNA methylation showed that all the noncoding regions are heavily methylated, whereas about half of the coding regions are clearly unmethylated. But under certain circumstances, such as stress, repressive chromatin modifications can be lost provoking the IGS transcription by RNA polymerase II (Earley et al. 2010; Audas et al. 2012; Saka et al. 2013; Bierhoff et al. 2014). In contrast to highly conservative gene sequences, spacer sequences evolve rapidly, mainly through nucleotide substitutions as well as deletions, insertions, or duplications of DNA segments, thus being highly variable (Arnheim 1980). They are widely used for molecular systematics and species identification, but data on the organization of the rDNA repeat units in birds are very limited (Dyomin et al. 2017).

The repetition of individual motifs may create the preconditions for their isolation and study through cloning. Early studies were based on the accurate sequence analysis of rDNA-containing plasmids and cosmids (Gonzalez and Sylvester 1995; Gangloff 1996). The routine technologies, such as Sanger sequencing or Next Generation Sequencing (NGS), generating relatively short reads, cannot provide sufficient overlap of repetitive regions. Long tandem repeated arrays with complex inner high-order repeat structures, which include rDNA repeat units, cannot be assembled from short-read sequencing data. The development of long-read sequencing technologies, such as the Pacific Biosciences (PacBio) or Oxford Nanopore Technologies systems, has made it possible to explore rDNA sequences. A good example of the use of such approach is the deciphering of the complete rDNA repeat unit of the chicken *Gallusgallusdomesticus* Linnaeus, 1758, which has only recently been made possible by PacBio sequencing of a BAC clone with an rDNA insert (Dyomin et al. 2019). The structure of the chicken rDNA differs from that of rDNA in human, apes, clawed frogs or fish, due to the highly GC-rich (up to 80%) spacers and internal repeats in the IGS (Dyomin et al. 2016, 2019). To date, the chicken rDNA repeat remains the only fully annotated avian rDNA despite the growing number of genome sequencing data from various bird species based on all existing sequencing platforms. The complexity of working with avian rDNA is that they are very rich in GC pairs, at least in ITS1 and ITS2 (Dyomin et al. 2017), and conventional targeted sequencing with site-specific primers is practically impossible even using reagents aimed at sequencing GC-rich templates. The complex structure of high-order repeats within the IGS that we discovered earlier (Dyomin et al. 2019) may lead to the possibility of complex secondary structure and intermolecular connections, which, in combination with GC enrichment, may be the reason for the depletion of data on these DNA regions during whole-genome sequencing. Extending the list of bird species with a sequenced and annotated rDNA repeat units is important not only for the development of taxonomic studies like to other systematic groups of creatures, but also for understanding the modes of karyotype evolution involving the nucleolar organizer region.

*Coturnixjaponica* Temminck et Schlegel, 1849 (Japanese quail) is a domesticated bird, widely used in the poultry industry for the production of meat and eggs. It also serves as a model species in biomedical, physiological, and embryological research, due to its small body size, short generation interval, and high fecundity (Lyte 2021; Strawn 2020; Molele 2022). *C.japonica* is closely related to *G.gallusdomesticus*, belonging to the same family Phasianidae, order Galliformes. These two species separated ~35 Mya (van Tuinen and Dyke 2004). They have the same chromosome number 2n = 78, no interchromosomal rearrangements have occurred (Takagi and Sasaki 1974; Schmid et al. 2000; Shibusawa et al. 2001, 2004; Kayang et al. 2006; Zlotina et al. 2012). An interesting difference from the standard Galliformes chromosome-evolution model is the finding of three nucleolus organizers loci in Japanese quail microchromosomes in contrast to the single locus in chicken and turkey (Kretschmer 2018; McPherson et al. 2014). Two pairs of microchromosomes bearing terminal nucleolar organizers remain unidentified, information about their composition is completely absent from the chromosome-level genome assembly data for Japanese quail (GenBank accession: GCA_001577835.2) and molecular markers for them are not described. One of the loci is situated on the Japanese quail microchromosome orthologous to chicken chromosome 16 (GGA16), bearing genes of the major histocompatibility complex (MHC). It was specified that the MHC-B and nucleolus organizer were localized at the q-arm. The MHC-B was more telomere proximal than the nucleolus organizer, similar to the genetic arrangement found in chicken. Furthermore, the q-arm is a DAPI-bright region, indicative of GC-rich DNA (McPherson et al. 2014). In the Japanese quail genome assembly *Coturnixjaponica* 2.1 sequences related to nucleolus organizer are absent: CJA16 (orthologue of GGA16) comprises 344 kb and contains dozens of annotated genes not corresponding to rDNA. Thus, the aim of this study was to decipher rDNA repeat unit sequence in order to find conservative and nonconservative elements in the IGS of two bird species and to understand the evolutionary dynamics of the rDNA in birds. We took advantage of the Oxford Nanopore sequencing technology to overcome the problems associated with the high GC enrichment of rDNA and the abundance of internal repeats.

## ﻿Material and methods

All manipulations with live animals and euthanasia were approved by Saint Petersburg State University Ethics Committee (statement # 131-03-2, issued on June 1, 2017).

Total genomic DNA was isolated from red blood cell nuclei of a Japanese quail female (Estonian breed) using standard phenol extraction procedures (Sambrook et al. 1991). The quality of the samples was assessed by capillary electrophoresis on a Qsep1 (BiOptic Inc., USA) device. DNA quantification was performed using a Qubit 4 fluorimeter (Thermo Fisher Scientific, USA). The libraries of the total genomic DNA were prepared using the NEBNext reagents (New England Biolabs, USA): NEBNext End repair / dA-tailing Module (E7546), NEBNext FFPE Repair Mix (M6630), NEB Blunt/TA Ligase Master Mix (M0367) following the manufacturer’s protocol. We generated Oxford Nanopore long reads on the MinION device operating with MinKNOW nanopore sequencing software using SQK-LSK109 library preparation kit followed by sequencing on a SpotON Flow Cell (R9.4) Single (FLO-MIN106D) according to the manufacturer’s instructions (Oxford Nanopore Technologies). The use of barcoded adapter BC02 (TCGATTCCGTTTGTAGTCGTCTGT) from Rapid Barcoding Sequencing Kit (SQK-RBK004) allowed the use of 1/10 GC-rich avian DNA samples in the flow cell load and improved the proportion of reads with complex secondary structure despite an overall decrease in coverage. The longest continuous raw reads containing in part or in whole the Japanese quail rDNA repeating unit (Suppl. material 1) allowed for the first time to obtain data on its organization, including the sequence of IGS, and to clarify sequencing and basecalling errors, as well as for sequence annotation, we performed additional alignments with all available data.

We began our bioinformatics analysis by searching for highly conserved sequences 18S–28S rRNA sequences based on data form chicken (Dyomin et al. 2016) in publicly available data of Japanese quail sequences and assembly data on NCBI database from the PRJNA292031 project (Nishibori et al. 2001). The fishing was carried out by using conserved sequences from the chicken rRNA gene cluster as the query. The contig derived from the incomplete Illumina read data was used to identify target data among the raw nanopore reads. Guppy (version 4.2.5) software was used for advanced basecalling and Minimap2, a versatile pairwise aligner for genomic and spliced nucleotide sequences (Li 2016), was used to mapping reads and de novo assembly.

Selected raw nanopore reads were converted into fasta format using the AWK command-line script (Free Software Foundation, Inc.). Using the fasta files as the database for BLAST and the 18S–28S sequence of *C.japonica* as the query we created a separate file containing .fastq nanopore reads that have similarities with the quail rDNA genes. Finally, we used SPADES 3.15.3 with default parameters to assemble all these sequences into contigs (Meleshko et al. 2019). To verify the validity of this approach, we performed assembly of the original nanopore reads without filtering using SPADES with the same settings.

To validate our results, we used publicly available *C.japonica* whole genome sequencing data obtained on Illumina next generation sequencing platform (NCBI SRR2159508). We filtered the reads using the TRIMMOMATIC tool (Bolger et al. 2014). We then used the contigs obtained in the previous steps as reference sequences and aligned all Illumina reads using the BWA tool (Li and Durbin 2010). The corresponding reads were extracted using the SAMTOOLS package (Danecek et al. 2021) and de novo assembled using SPADES. Finally, we used GATK4.0 Haplotype Caller algorithms (Poplin et al. 2021) to check the corrections made by the Illumina data. rDNA sequence annotation was performed using GENEIOUS 9.1 (http://www.geneious.com).

Sequence annotation with gene boundary determination was performed based on conserved element data. Determination of the location of the promoter and transcription start site was accomplished based on transcript mapping from RNA-seq data (NCBI SRX9608520, SRX9608583, SRX574377) that were downloaded using SRA-toolkit (http://www.sthda.com). The reads were trimmed using the TRIM_GALORE program (https://www.bioinformatics.babraham.ac.uk). Mapping reads were conducted using BOWTIE2 (http://bowtie-bio.sourceforge.net). The resulting SAM file was converted, into a BAM file sorted from duplicated rows using SAMTOOLS (http://samtools.sourceforge.net). Then we converted BAM file to WIN format using the following script: https://github.com/MikeAxtell/bam2wig. The sequence coverage was calculated using SAMTOOLS. We visualized the coverage and found the starting point of transcription using IGV browser (https://software.broadinstitute.org) (Suppl. material 2).

## ﻿Results

Because rDNA is typically underrepresented and fragmented in avian whole genome sequencing data, we first turned to transcriptome sequencing data, where ribosomal RNA is expected to be represented, to obtain primary information. Among Illumina reads from the PRJNA292031 project (Nishibori et al. 2001) we selected 510 reads by alignment to a reference chicken rDNA based sequence containing conservative rRNA regions. They were assembled into a three contigs with the length of 1065–3679 bp overlapping 18S–28S rDNA region, which was used to search for target reads in the following sequencing data obtained.

Using Oxford Nanopore Technology whole-genome sequencing we obtained 36524 nucleotide sequences, 74 of which were then aligned with 18S–28S rDNA region. The length of the aligned sequences ranged from 297 to 14126 bp. These sequences were then assembled into 13 contigs one of which had a length of 19964 bp, and included partial sequences of ETS and complete sequences of 18S–28S rRNA. After the pairwise alignment of each of the 74 reads with 18S–28S rDNA the sequence with length of 21166 bp which covers complete IGS region was obtained.

The contigs we obtained from this project were then aligned to three Illumina based contigs with the length of 1065–3679 bp. The comparison showed a high degree of similarity between 5’-ETS-28S sequences and the Japanese quail rDNA de novo assembly. We found 88.7% similarity to the 2,350 bp long region corresponding to 5’-ETS-ITS1 (completely overlapping the 18S rRNA gene), 90.3% similarity to the 1,085-bp-long region containing ITS1, and 88.4% similarity to the 3,735-bp long region of the 28S rRNA gene. These percentage indicates high quality of the consensus sequence obtained. Nevertheless, the 18S, 5.8S and 28S rRNA genes were edited according to the Illumina sequences.

The resulting 21166 bp sequence was accepted into GenBank under the registration tag BankIt2509210 CoturnixOK523374. The *C.japonica* rRNA genes boundaries were identified by comparison with the fully annotated sequence of the chicken *Gallusgallus* (MG967540). Comparison of the contigs we obtained with raw data from an alternative source (ERR11591487 and ERR11591488, French National Institute for Agriculture, Food, and Environment) showed the presence of raw reads with high homology – up to 90.6% and coverage – up to 99%.

*C.japonica* rDNA repeat unit was described through comparison with the rDNA repeat of the chicken, completely annotated previously (Dyomin et al. 2016, 2019). It includes 5’-ETS (1779 bp), 18S (1823 bp), ITS1 (2047 bp), 5.8S (157 bp), ITS2 (658 bp), 28S (4185 bp), and 3’-ETS (639 bp) followed by IGS (9878 bp) with the total length of 21166 bp (Fig. 1).

**Figure 2.**
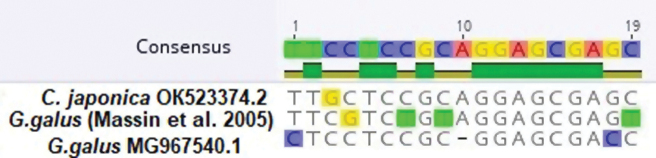
rDNA promoters operated by RNA polymerase I.

The 5’-end of the 5’-ETS was determined by finding the transcription start point, using RNA-seq data from the NCBI (Suppl. material 2). The obtained RNA polymerase I promoter region of *C.japonica* rDNA (TTGCTCCGCAGGAGCGAGC) was compared with a similar chicken sequence MG967540, as well as with the one described by P. Massin and co-authors (Massin et al. 2005). The differences ranged from 1 to 4 nucleotides with a promoter length of 19 nucleotides (Fig. 2).

The primary sequence of the *C.japonica* rDNA genes - 18S, 5.8S and 28S - turned out to be conservative and demonstrates a high degree of similarity with the corresponding chicken regions. The determined Japanese quail rDNA gene sequences were 99.5% identical for 18S, 100% identical for 5.8S, and 85.7% identical for 28S described earlier for chicken. The sequence of the 5’ETS is highly GC-rich (74.1%), which is comparable with the chicken 5’-ETS (74.9%). The number of dispersed inverted repeats (CGG)_92_ and (GCC)_78_, as well as (GCGA)_22_ and (CGCT)_10_ was 28.4% and 7.1%, respectively. In addition, a direct microsatellite repeats (GTGCC)_4_ and probably a degenerate repeat (CAGM)_5_ are represented in the 5’ETS region: CAGACAGGACAGACAGGCAGA. ITS1 sequence (2047 bp) has high GC content - 76.8%, as well as ITS2 (658 bp) - 78.0% GC, which is higher than in 5’-ETS and 3’-ETS. However, compared to chicken corresponding regions, the GC content in each of them is lower (Table 1).

**Table 1.** Comparative characteristics of the CG composition of quail and chicken rDNA.

	5’ETS	3’ETS	IGS	ITS1	ITS2
	CG (%)				
*Coturnixjaponica*	74.1	72.1	66.3	76.8	78.0
*Gallusgallus*	74.9	79.4	69.2	82.1	82.2

**Figure 3.**
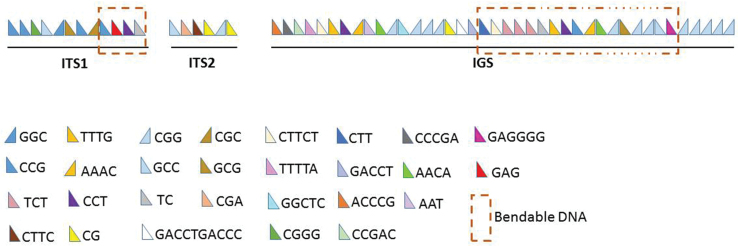
Distribution of repeating elements in *C.japonica* ITS1, ITS2 and IGS.

The dispersed inverted repeats in ITS1 were represented by (CCG)_12_ and (GGC)_3_, (GCG)_3_ and (CGC)_3_; in ITS2 - (GCC)_3_ and (CGG)_6_. Besides, there are several direct microsatellite repeats in ITS1: (CGGG)_3_, (GCC)_3_, (GAG)_3_, (CCT)_3_, (TC)_5_ and in ITS2: (CGA)_3_, (GTTC)_4_, (CG)_6_, (CG)_5_ (Suppl. material 3: tables S1, S2). The repeating elements in ITS1 form extended polypurine / polypyrimidine bendable tracks that have the potential to form non-canonical secondary DNA structure, in particular the H-form (Fig. 3). The size of the 3’-ETS (639 bp) was determined by the localization of the transcription terminator, namely the poly-T region followed by inverted repeats. The boundaries of the second external transcribed spacer were determined this way, since transcriptome analysis demonstrated transcription attenuation on the border of the 28S gene. The CG content was 72.1%. In chicken, the same region contains 79.4% GC (Table 1). The dispersed inverted repeats are represented by (CGG)_17_ and (GCC)_14_. At the terminal part of this non-transcribed spacer, about 100 bases long, the following direct repeats (microsatellites) are found: (ACCCG)_5_, (CCCGA)_3_, and (CCGAC)_3_. Probably, the last two represent a single sevenfold repeat with cytosine drop out at two positions: CCCGACCCGACCCGA**CCGA**CCCGACCCGA**CCGA**.

The Japanese quail IGS includes 9878 bp containing twenty tandem repeats (Suppl. material 3: table S3). The GC content in this region is 66.3% versus 69.2% in chicken (Table 1). In addition, the repeat (GACCC/T)_10_ is probably degenerate because of the alternating nucleotide or there are two different tandem repeats (GACCTGACCC)_3_ and (GACCT)_4_. The extended non-transcribed region of the IGS is enriched in polypurine / polypyrimidine sequences prone to bend causing the formation of non-canonical secondary DNA structures (Fig. 3). The repeat element (GAGGGG)_n_ may also contribute to the formation of the G-quadruplex.

## ﻿Discussion

To better understand the biological processes occurring in the rDNA and the nucleolus, their common and particular aspects due to the physiology of a given species, reference rDNA sequence should be developed for every taxonomic group: human rDNA for Mammalia, *Xenopus* sp. for amphibia, *Daniorerio* F. Hamilton, 1822 for fish, terrapin for turtles and crocodiles. Most bird species are characterized by the presence of one pair of chromosomes carrying the nucleolar organizer regions with an ancestral interstitial localization. Despite advances in sequencing avian genomes using various platforms in recent years, most available datasets fail to provide the sequences of the rDNA repeat unit suitable for comprehensive analysis. Before this work, the rDNA sequence of only one bird species had been deciphered – the rDNA of the chicken (Dyomin et al. 2019), so we compared our new results with it. The sequence of chicken ITS1 is longer than that of most animals, with few exceptions (Coleman 2013). A similar region of the Japanese quail genome was found to be 401 bp shorter. *C.japonica* ITS2 compared to the those of the chicken is also smaller. In this case, the difference was 76 nucleotides. Internal transcribed spacers are used as a convenient marker in phylogenetic studies. The recently described fact of the complication of ITS1 and ITS2 sequences in the evolution of Deuterostomia, the accumulation of GC nucleotides, and the elongation of sequences are surprising and currently unexplained (Dyomin et al. 2017). As defined by M. Gardiner-Garden and M. Frommer (1987), we assume that any stretch of DNA greater than 200 bp with a GC content greater than 50% and an observed to expected CpG ratio greater than 0.6 is a CpG island (Gardiner-Garden and Frommer 1987). A comparative analysis of Japanese quail and chicken rDNA repeat unit showed the comparable level of CpG island retention - 83.3% in rDNA of *C.japonica* and 89.7% in rDNA of *G.gallus*.

To date none of the regions of the quail rDNA cluster containing tandem repeats enriched with polypurine / polypyrimidine tracts of bendable DNA and extended repeats was represented in NCBI databases. According to our data (Fig. 3), *C.japonica* ITS1 and IGS are saturated with such elements to an even greater extent than the corresponding regions of the chicken rDNA repeat unit. Due to features of the secondary structure with the potential to form H-isoform DNA with sticky single-nucleotide arrays, bendable DNA of the polypyrimidine / polypurine tracts may play an important role in nucleolus formation involving nucleolus organizers from different chromosomes. We paid special attention to scattered inverted repeats, since RNA transcripts usually fold to form hairpins of different lengths (Singer and Berg 1998).

Functional state of such intracellular structures is one of the qualitative criteria of the physiological state of a cell as a whole. Along with the main function of ribosomal gene clusters - rRNA synthesis for ribosomes - there are now secondary or noncanonical functions associated mainly with transcription-inactive rDNA. A decrease in the number of inactive copies leads to instability of the entire nucleus chromatin, increases the sensitivity of the cell to damaging influences, and promotes accelerated cell aging (Paredes et al. 2011; Kobayashi 2014). It is known that nucleus can accumulate proteins which do not take part in ribosome biogenesis. The ability of rDNA copy to transcribe depends on its conformation and epigenetic modification - cytosine methylation in the CpG site (Ershova and Konkova 2020). Modern cytogenetic methods allow detection of such rearrangements and objective estimation of the activity degree of nucleus-forming regions of chromosomes. Morphological variants of the nucleus in tissue samples (including metastasized ones) with cancer dysplasia characterize the degree of functional activity of cells and reflect the level of processes related to ribosome biogenesis (Bolgova et al. 2012). Changes in functional state of loci due to such processes as activation or repression of transcription, DNA damage, and cell differentiation can be accompanied by relocation of this locus and large-scale changes in the entire genome architecture. However, the data obtained using karyotype analysis methods is insufficient for a complete understanding of the mechanisms of transpositions and chromosomal rearrangements. Analysis of the primary rDNA sequence provides an opportunity to study not only the consequences, but also the sites responsible for such rearrangements - transposons and other mobile elements.

The best studied rDNA repeat unit is the human. It is about 43 kb, there are 100–500 copies in a cell (Agrawal and Ganley 2018; Parks et al. 2018). The IGS comprises three copies of the R repeat containing the Sal box terminator sites (Grummt et al. 1986). At the center of the IGS there is a repetitive region composed of a Long repeat, CT microsatellite, and Butterfly repeat. Some elements associated with non-coding RNA working in stress response, a cdc27 pseudogene, and putative c-Myc and p53 binding sites as well as conserved sites with unknown function have been annotated (Gonzalez and Sylvester 2001; Agrawal and Ganley 2018). The organization and the degree of divergence of the human rDNA units were studied using FISH, which allowed revealing rDNA units which were non-canonically oriented (Caburet et al. 2005). Deciphering of the entire individual nucleolus organizers on chromosomes 21 and 22 (Kim et al. 2018, 2021), studying variation by nanopore sequencing (Hori et al. 2021; Wang et al. 2023) evidenced the concerted evolution of rDNA units.

The Japanese quail genome as a model provides a good opportunity to study the specific processes occurring in the nucleolus organizer region related to genome activity of repeated DNA and, as a consequence, leading to the high risk of chromosomal rearrangements. Nucleolar organizers in *C.japonica* karyotype are localized on three different pairs of chromosomes. In addition to the ancestral interstitial nucleolus organizer on chromosome 16, there are two more terminal nucleolus organizers on the short arms of the acrocentric chromosomes (Solinhac 2010). We have not found any sequence variations in the primary ribosomal gene sequence, which may indicate characteristic features of rRNA gene clusters localized on different particular chromosomes. The previously shown involvement of transposons in the relocation of rRNA gene clusters to the terminal sites of the two microchromosomes may explain the lack of significant sequence changes in the clusters themselves (Saifitdinova et al. 2019). To identify the features of chromosome-specific nucleolar organizers, it is necessary to increase the collected amount of data based on long-read sequences that are more tolerant to high GC content and to improve methods for overcoming the difficulties caused by the presence of sticky regions enriched with repeats prone to the formation of non-canonical DNA forms and intermolecular connections.

Advanced techniques may finally allow the identification of two of GC-rich microchromosomes saturated with tandem repeats carrying terminal nucleolus organizers, which are still not represented in *C.japonica* chromosome-level genome assembly (GenBank accession: GCA_001577835.2), for which there are no molecular markers and no defined gene linkage groups. Knowing the characteristics of avian rDNA based on the previously studied chicken ribosomal cluster, in this work we used the possibilities of barcoding and diluted the Japanese quail genomic DNA sample with non-targeted DNAs with a lower GC content to reduce the likelihood of adhesion of the desired molecules. Although the total number of reads and the coverage was not very high, we were able to obtain data on the *C.japonica* rDNA sequence for the first time. In this work, using nanopore long-read sequencing we identified and validated with NGS dataset the 21166 base pair of the complete Japanese quail ribosomal gene cluster sequence (GenBank under the registration tag BankIt2509210 CoturnixOK523374). This is the second deciphered avian rDNA cluster after the chicken, with some similarities to it as well as characteristic differences.

## Supplementary materials

Supplementary material 1 The longest obtained raw nanopore reads containing Japanese quail rDNA repeat unit sequences Data type pdf https://binary.pensoft.net/file/1140974This dataset is made available under the Open Database License (http://opendatacommons.org/licenses/odbl/1.0/). The Open Database License (ODbL) is a license agreement intended to allow users to freely share, modify, and use this Dataset while maintaining this same freedom for others, provided that the original source and author(s) are credited.Alina Zhukova, Gennadii Zakharov, Olga Pavlova, Alsu Saifitdinova

Supplementary material 2 Localization of the promoter and transcription start site using RNA-seq data mapping Data type pdf https://binary.pensoft.net/file/1140975This dataset is made available under the Open Database License (http://opendatacommons.org/licenses/odbl/1.0/). The Open Database License (ODbL) is a license agreement intended to allow users to freely share, modify, and use this Dataset while maintaining this same freedom for others, provided that the original source and author(s) are credited.Alina Zhukova, Gennadii Zakharov, Olga Pavlova, Alsu Saifitdinova

Supplementary material 3 Composition of tandem repeats in the noncoding spacer sequences of Japanese quail rDNA repeat units Data type pdf Explanation note **table S1**: Characteristics of the Japanese quail ITS1 tandem repeats; **table S2**: Characteristics of the Japanese quail ITS2 tandem repeats; **table S3**: Characteristics of the Japanese quail IGS tandem repeats. https://binary.pensoft.net/file/1140976This dataset is made available under the Open Database License (http://opendatacommons.org/licenses/odbl/1.0/). The Open Database License (ODbL) is a license agreement intended to allow users to freely share, modify, and use this Dataset while maintaining this same freedom for others, provided that the original source and author(s) are credited.Alina Zhukova, Gennadii Zakharov, Olga Pavlova, Alsu Saifitdinova
