# Supplementary material for: ﻿Description of the complete rDNA repeat unit structure of Coturnixjaponica Temminck et Schlegel, 1849 (Aves)
Source: Comp Cytogenet. 2024 Sep 25;18:183–98. doi: 10.3897/compcytogen.18.127373 (PMC11447458; doi:10.3897/compcytogen.18.127373)
Supplement: Supplementary material 1 — The longest obtained raw nanopore reads containing Japanese quail rDNA repeat unit sequences [file comparative_cytogenetics-18-183_article-127373__-s001.pdf]

>14.95.eb29ae5a-4a45-4721-babb-813897bfde4d protocol_group_id=2020-02-09_1st ch=289 barcode=BC02 read=3375 start_time=2020-02-09T17:03:07Z flow_cell_id=FAK79217 runid=88172aec3f27372c4684111da770399a9fd1fb40 sample_id=2020-02-09_1st

CTGGCGGGCCGGCTCAATCCCTCCCGTGTCGTTCGGGCGGCGTGGGAGATGACCGGTGTTCAGGCACAGGGCGA
GCACCTCTCGAGCGGGCGTTGCCGCACACCCCACCGCACTTGCGTGCGCGGTCTTTCACCCACGCGCCCGGGTGG
AAGGGCTGGTACCTTCCCCACCCCGTCCCTCCCCTCTTCTACCTCACCGCACCCTTACCCCTCCCGTCCTCCTGCCTC
CCCCTTCTTCAAGTGATCGGTAAGGGCCATTCGAGATCGCGTCGGTGAGAGACCCCGACCAGGCCAGGCGCTTCT
GCGTTCCCCGTCCCGGGGAAGCGCTGCGGCGTCGGTGTTCAGGCGCGCGAGGCGGTCTCCTCTCGGTTCGCTTCC
CGTCGTACGCGGGTGAGGCGCTCTCCCGGCTCGCTCAGAGCGGCCGAGCTTCGCCCGGTGAGCCAGTGACGGCC
GAGCGGCCCAGCGAGCCGCGGTGTGCCCAGTGAGTCGTGAGCAGGCGCCGGCGCCGACCCTGTTCGAGACCGAC
GGGGAGTGCCGGCCCACTTGAGCGCTGCAAGAGCCGGCAGGGTGTCAAACGAAACCGTCCGTTCGAAGCGAGGC
GAGAACTAAAGTTAGGAGCGGGGGCGTCGCCGGCCTCCCCTCCGCCCGCCCGGGCCGCTGGGGCCCGCGTGCGT
GGGGTTTCCCTTCCGACGTTCCCGCCCGGCTGCGGGCGTGCCGCCTCGGGAAGAGGGTCTCCGGTGTTGGGGCG
GCGTCCGCCTCGAGGTGCGCCTTCTCCTCTAGCGCGTCCGCCAGTCTCAGCAGCGGGGTTCCGTTCCCTTCGCCGC
GGTCCGGAGGAGCATCGGGTCAGCCCGGCCGGTGCCGCCGTGCCGTGCCGTGCCGTGCCGCGCCGGCTCCGGCG
TGAGCCGGGCGCGCCGCCGGCAGAGAGCGCGCGACGACGCGAGTGCTGCGCAGCGCGCCCGCTCCTTCCCGCAT
GGGGAGCGCGCGGAGTCCGCCTTCGAGGAAGCAGGCGCGCCTCTCCGCCCCGCCCGGTCCGCCCGCGCGGCAGC
GTGGCCGGCGCGGAGGGCCGCCCCTCCCCCGCCGAGCGACCCGCCCGTCAGCGCCGCCGTCGACCCGCAGGAAG
GACGTTAGGGGCAGGCAGTGGCTCGAGCCAGCCAGTCAGCCGGTTGGATCCCGAGGCGAAGAGAGGGCGCGGT
TGAGCGGTGGGCGTCGGCGAGTCGGGCGCGTCGGGTCGTCGCCCTTCCAGGCGCGGCTACCTGGTTGATCCTGC
CAGTAGCATATGCTTGTCTCAAAGATTAAGCCATGCATGTCTAAGTACACGGACGGTACAGTGAAACTGCGGAGA
TAGCTCGATAAATCAGTTATGGTTCGCTTTGGTCGGCTCCCTCCGTTCCTTGGACTAACTGTGGTAATTCTAGAGCT
AATACATGCCGACAGCGCCGACCTCAGGGATGGCATTAGAGTTTTATCAGACCAAAACCAACCCGGGCTCGCCCG
GCAGCTGGTGACTCTGGGTAACCTGGAGCGATCGCCGCCCCGCGGCGGCGAAGCGACCCATTCGAATGTCTGCCT
ATCAACTTTAAATTGGTTTTGTCTGTGCCTACCATGGTGACCACGGGTAACGGGGAATCAGGTTCGATTCGGAGAG
AGAACTGAGAGCAGCTACCACATCCAGGAAGGCAGCAGGCGCGCCCAGAGTACCCCTTCCGACCGGGAGGTAGT
GACGAAAAATAACAATACAGGACTCTTCGAGGCCCTGTAATTTAAGAATGAGTCCACTTTAAATCCTTGAGGATCC
ATTGGAGGGCAAGTCTGGTGCCAGCAGCCCGCGTACTTGGTAATTCCAGCTCAATAGCGTATCTTAAAGTTGCTGC
AGTTAAAAAGCTCATGTAGTTGGATCTTGGGATCGAGCTGGCGGTCATAGCAGTGACCATCTGTCCCAGCCCCTGT
CTCTCGTATTACTCGATGCTCTTAAGTGAGTGTCCCGCAGGGCCCGAAGCGTTGCGAAAAATTAGAGTGTTCAAAG
CAGGCCGGCCGCCCAGGAATACTCCAGCTAGGAACTTAATGGAATAGGACTCGGTTCTATTTTATTTAGTTTTTGG
AAGCTTGGGGCCATGATTAAGAAGGAAGGCCGGGGGCATTCGTATTGTGCCGCTAGAAGGTGAAATTCTTGGAC
CGGCGCAGGAACGAACTAAGCAACATTTGCCAAGAATGTTTTCATTAATCCAGAAACGAAGTCGGAGGTTCGAAG
ACGATCCAGATACCGAAATGGCCAGTTCCGACCATAAGCGATGCCGACTGCGATCCGGCGCGTTATTCCATGACCC
GCCCGGGCAGCTCAGAAACCCAAGTCTTTGGGTTCCGAAGTATAATTGCAAAAGCTGAAACTTAAAGGAATTGAC
GGAAGGGCACCACCAGAGTGGAGCCTGCGGCTTAATTTGACTCAGCACGGGAAACCTCACCGGCGGACACGGAC
AGGATTGACAGATTGAGAGCTCTTTCTCGATTCCCGTGGGTGGTGGTGCATGGCCGTTCTTAGTTGGTGGAAGCG
ATTGTCTGGTTAATTCCGATAACGAACGAGACTCTGGCATGCTAACTGAATTCTAGCAGCCCCCGAGCGGTCGGCG
TCCAACTTCTTAGAGGGACAAGTAGGCGTTCAGCCACCAGATGAGCAATAACAGGTCTGTGATGGCGGCCCTTAG
ATGTCGGAGCTGCACGCTACACTGACTGGCTCAGCTTGTGTCTACCCTACGCCCGGCAGTGTAGGGTAACCGTTGA
ACCCCATTCGTGATGGGGATCGGGTGCAATTATTCCCCATGAACGAGAATTCCCAGTAAGTGCGGGTCCTTAAGCT
CGCGTTGATTAAGTCCCTGCCCTTTGTACACACCGCCCGTCGCTATTTACCCGATTGGATGGTTTAGTGAGGGTCCT
CGGATCGGCCGGCGTATCGTACAGGCTGTACGGAGCGTCGAGAAGACGGTCGAACTTGACTATCTAGAGGAGTA
AAAGTCGTAACAAGGTTTCCGTAGGTGAACCTGCGGAAGGATCATTACCGGGAAAAGCCGAGGATGGAGCGACC
GGAGTCGGTCTCCCTCCGCCTTCGGTTCTTTCGTTCCTTCTTCCTTCCTTCCTTTCCTGACGCGCTCTTAAAGCGGGC
CAGAGCTCGAAACAGCCAATGCCCGAAGCGCGGCGCAGCGAGTCACTCCGCACCCCCATGAGACGAGAGAGAGA
AAGGGGCTGCGGGTCGCGGGCGCGGCCGCGGGGGCGGCGTGGGGGAGGCGAGTTGGAGGCTAGGGAGGGAG
GAAGGAAGGAAGGGGCAAAGAATTGTCGTCGCGCCGGGGCGGAGCGCACCCTCGCTGCGCCTTTCCTCCTATCCT
CCCCACGGCTTCCCTTCTCCCTCCCCACGAGCTCTCCGCGCTGCGCGGTCCGCCGCCCGGTCCGGTCCGCCTCCGGA

GTCGGGACCGTCCGCCGGTCGCCTCGCCCCGCCGCTGGGGCGCGCAAGCGCGTGGCTCCCGTGGCCGGCCCGGT
CCCAGCAGCGGGCGGGGCGGTCCGAGCCCGCGCGTTCCTCCCGGGCACAGCTGCCGCCGGGCGCCCGGGTTACA
GAGAAACCAGGGCCGGGAGAGAAGCCGAGTGGCGCCGAGACGGCAGGCGCGCGCGCCCCGTAGTAAGACAGCT
CCCCGAGGCGCGGGGCCGGCTGAGTGCCGGGTTTCCCCTCGGCGCGCCGTCCCGTGCCGTGCCACCGGCCTTCGG
CCGGTCGGCCGGGCGGGTGGAGGGACCCCCCGCGGGGTGGGGTCGTTTCCCTCAGCCCAGGGCCCGGTGCCGC
GCCAGCGCCTCACCAGGCAGGCGGAGGTTTAAAGACTCCGAAGAGCGGCCCGCGGCTCGCTCCGGCGAGGGAAA
GAAGGAGGGCCGGCGGCGCTCCGGCGGACAACGAGGAAGAGGAGGCTCAGCGGGCGCGCGGCGCCTCCGCCC
CTCTCCACGAGCCACCGGGTGCGATCGCCCGCCGCGCTCCCGGCGGTCAAACCCCCTCCCCACGCTGCGGCCGAG
GGAGTGCCGGGCCGGAGAGGGGTTGTCCCGTCCCCCTCTCGCGGCGTCGGTCTCGGCGGAGGAGTCCGGTGCGG
CGCGAGGCGCGCCGCGTCGCGCTCCGGCCGGCCTCGGCCCCGCGTTCGGGCGGGCCGAGGCAGTGTCTCGGCCG
GCGCCCACCCGCTCCCCACCGCGACGGCGAGCGCCGAGTGCGTCAGCGATCGCCTCCCCGCCGTGCGCGCGCGGT
AAAGTGCGTCGGCAGCGTCCGCCGCGCCCTCGCGCGATCCCCTTCCCCGGGTCCGCGCCACGCCGTTTCCCTCGGC
GTCGCGAGCCGCGTGCGTCGTCGTCCCGCCTTCGCCGCTCTCCACGCCTTTGCCTCGGCCTCGCCCGGCACCGCGC
CCGTCGTGCAGCGGAGGTCGGCGTGAGGCTGTTGCAACCCGGTCTCGTGCTGGCGCGGGGGATGACGCCGCTCT
AGATCCGTCCCGTCCGCCCCCGCCGCCCGGTCGCGAGTCCCGCTGCGAAGCGCGCGCCAGAAGCGAGAAGAGAA
GGAGAGCGGCCCGGAGGTCGAGGACCGAGGCGGAGGCGGAGGCGTCGGGGAGTGCGCTTTGGGGCGCTTTTG
CCAGTGGGGCAGCTGTGGAGGGTCTTTGCCGAGGCGGAGCAGGCGCTGCCGAGGCGGTGGTCGGCAGCGATCC
CGGCGCGTCAGGCTGGGCTTTTGCCGGGCGCGCGTGCCGTCCCAGCGTTACGTCAGCGGCCTCCTGTCGGGCAGC
CGAGGCAGGCGCCCTGGTCCGTCCAGCGAGACAAGTCGTTCCCAGGTCGGGAGCGGGGGCTCCCCGCCCCTCGT
TCGGGGTGCGCTATGTTTCTCTCCTCTCCTTCGATGGTGTGCTCGTTAGCGGTCTCGCCCGGAGAGCGTGCTCGTC
GCCCGTCCGCTCCTTGGTGGTGATCCGGCCGTCCCGGGCGCTCGCGGTGCGGTGCGATAGCAGGGCCGCCTTCCT
GGGTGGAAAGCGGCGAAAAAATCGAGAACTCCCAGCGCGAAGCGGTGCCGAGATCAGACAACTCTTAGCGGTG
GATCACTCGGC-CGTGC--CGATGAAGAGCGCAGCTAGCT-CGCGAGGTAATGG----
TGCAGGACACATTGATCATCGAC---CCGA--GCACTT-CGGC-
CCAGGTTCCTCCCGGGGCTGCACGCCTGCCACCTGAGCGTCGCTTGACGGTCAATCGCCGTGCCACCGTTGCGCG
GCATCATGTGAGCGCGGCTGGGCGCCTCGCCGGGCCCGCGCGCCTCCGGCGGCGAAGGTCGAGGGTGCGCGGC
GGGCCTTCGTCCCCCTAAGTGGAGACCCCAGGTCGGGGGCTCGCTGGGCGAAGCTCCCAGCGCTCGGGCGCCCG
CTCTGGCCCGACCTCGTCCCAGCGGAGTCGGGCTCTTCCGATCGATCGCGCCGCGGGTCCGGTCCTGTTCGTTCGT
TACCGTTCGCCCTTCCCCGCGTGCGTTCGTTCGTTCGGCGTCCGGCCCCCGGCCCGGAAGGAGAGGCCAGTCGTC
GCCCTCCCGGCCGGCGCGCGTCTGCCCTGCCCGGAGTCGCGTCACCGGCGGCGGTGTCATGCCCGTGCTACCAGC
GAAATTTCAGGCCTTGATTTCCAGCCCCTCCGGTCGTCGGTCCGTCAGCGTAAGCGGCGGCCCAGCGGCCGGCG
CTGCCGATCGCGGGTCGTCTCCGGCCCGCAGGGCGCGGCCTCGGCAGCGCGTCCCTTTCCTTCGCGTCAGGCGTC
GTCCTCCAGCGCGTGGCTTGGTGGGCTCGGGAGGGAGCGCGTCTCGGGGCTCCGCTCGGTGGAAACCCGTCGGG
GCGGGGCCCTCGGGATACGTAGCGACCTCAGGTCAGGCGTGGCGACCCGCTGAATTGCTTATATTAGTCAGCGGA
GGAAAAGAAACTAACGAGGATTCCCTCCCAGTAACGGCGAGTGAAGAGGAAGAGCCCAACGCCCGAATCCGTCC
AGCCAGGTGGGCGCGGGAGGTGTGAGTCTGAAGCCCATCCCCGAGCGCCGCTCTCGGGAGGCCCAAGTCCTTGT
GATCGGTATCCAGCGGGCGGTGGCGGGCGGTAGCGGCCCCCGGCGCGCCGGGCCGGGGGCTTCGAGTCGGGTT
GCTTGGGAATGCAGCCCAAAGCGGGTGTAAACTCCATCTAAGGCTAAATACCCAGCCTGAGACCGATAGCCAACA
AGTACCGTGAGAGAAAGTTGAAAAGAACTTTGAAGAGAGAGAGTTCAAGGAGCGTGGGGAAACCGTTAGAGG
TAAACAGGTGGGGTCCCGCGCAGTCGAAGCCCCGGAGGTTCAACCCGGCGGAGGCGACTCGGCCGGCCGGCGC
GGGCGCCCGTCCGGATCCCCCGCCTCCGCCTCCCCCTCCGCCCGCCCCTTCGCGGGGCCAGAGGCGGGCAGGGGA
GCAGGCGGGCCGGGGACCGCCACGCCGTCGCGTCCGGCCCCGTCCGGGCACAGCTCCTCCCGCGGCGATGCGCC
GCGGCCGGCGCTCCCGGGGCGGCGGGAGCCTCCGGCGGGCAGGTGGCCCGAGCGCGCGGCCCAGTCCCGCGGG
AGTTAGAGCCCGGGCCCGATCGTCGCCCAGAAATCCGGGGCGAGGATCAGACACCGATCCGCCGCCCTCCCCGG
AAGGTGGCGGGGTGCCTTGGGGAGCCTTACCCGCCTCTTCGCGCCTCCCCCCCGCCTTCCCTTCCTTCGCCCGGTTT
CGGGCGGGGAGTCGAGACGGGCCGCGGCCAGCGCCGGCGTTCAGGCGGGGGCGGACTGCGTAGTGCGCCCCG
GCGCCGCGGCGCCACCCGGGCGGGCTCGAGCCACGCTCGGGCGCTCGGGGTCGCGGCGACGTCGGCTACCCACC
CGTCTTGAAACACGGACCAAGGAGTCGACACGCGCGCAGTCGCGGCGGCTCGCGCGCCCGCGGCGCAATAGAAG
GTGAAGGCCGGCGCCCAGCGCCGGCCGGTGAATCCCAGGGCGGCGTGTCGCGAAGCCCCAGGCGCACCACCGG
CCCGTCGTCCCGCCCCGCCCGGGGCAGCGCGTGCGGGACGAAGATGGTGAACTATGCCTGGGCGGAGGCAGCCA

GAGGAAACTCTGGTGGAGGTCCGTGGCGGTCTGACGATGCAATCGATTCGTCCGACCCGGGTATAAGAGGCGAA
AGACTAATCCAGACCATCTAGTAGCTGGTTCAGTTTCCCCTCAGGATAGCTGGCGCTCGGGGGCGGCGGAGCAGT
TTACCCGGTCAGCGAATGATTAGAAGGCGTTGGGGCGAAACGATCTCAACCTATTCTCAAACTTTCAATGGGTAAG
ACGCCGGCTCTGCGGCGTGGAGCCGGGCCGTGGAGATACGAGCTGATGGGCCACTTTTGGTAAGCAGAACTGGC
GCTGCGGGATGAACCGAGCGCGGGTTAAGGCGCCAGCCCGATGCAGCGCTCATCAGAGCCCAGAAAGGTGTTGG
TCGATCCTACAGACAGCAGGGCGGTGGCCATGGAGTCGGAGCCGTAAGGAGTGTGCAACTCACCTGCCGAATCA
ACTAGCCCTGAAAATGGATAGCGCTGGAAGCGTCGGGCCCATACCCGGCCGTCGCCGGCGGTGCGAGAGCATGG
GGGGGCTACGCCGCGCCCGGCAGTAGGAGAGACATGCGGTGCGCCTGGAAGCCTGGGGCTGGCGGGCGGGTG
GGCCGCCGCAGGTACAGATCTGCTAGTGGTAGTAGCAACTATTCAGACAGGCTTTGAAGGCCGAAGTGGAGAAG
GGTTCCATGTGAACCTTGATTGAACATGGGTCAGTCGGTCCTAAGCGATGGCGCGCCGTTCCGGAAAGGACCGGG
CGATGGCCTCGAAATGCGCCCTCGGCCGGAAAGGGTGGGTTCAGATGCGAATCCCGGGCAGCGGAGACGGGGC
GCCGCAGGGCGCCCAGTGCGTGCCTTGAGCGTCCCGGAGAAGCCGGCGAGGCGGGGAGAGTTCTCTTTTCTTTGT
GAAGGGCCGAGGCGCCCTGGGCAGGTTCGCCCGAGGGGGCCCGCGCCTTGGAAAGCGTCGCCCGGTTCGGCGG
CGTCCGGTGAGCTCTCGCCGGCCGTGAAAATCCGAGGGGAGAGGGTGTAAATCTCGGCGCGGGCCGTACCCATA
TCAGCAGCAGGTCTCAAAGGTGAACAACTGGCGTAGACAACGTAGGTAAGAAGTCGGCAAGCCGGATCCATTAA
CCCGGGATAGGGTGGCTCTAAGGGCACAGGTCGGTCAGGCTGGGACGCGAAGCCCAGGCTGAGCGCGCGCTAC
GCTGGACCGAGGCGCGCCCGCAGGCCCCAGCAGCGCTCCTCCCCCCCGGCTCCCCCTTCCTTCCCTTCCCCGGCCG
GCGTTCCGGTCGGCGCGGCGGGGGGCGAGAGGCGAGGTCGGGGGCGCCGTCGGCAGGCGCGGCGGCGGCGGC
TCTGGGGACGCGCGCGCCGGAGCCCTTCGTGGATCGCCGCTGCGGCCCAGGCGCCGCCCGCCCCGCTTCCCTCCG
CCTCGCTCCGTCGCCCGTTTCCCGCGTGCGCGGGCGCGCGTGGCGAAGCGGCGGGTCCCGGGCCAGCGCCCCGC
CTCGGCCGAGCGCCTGAGCCGGCTTAGGAGCTGGTGCCCGGTTTGGGGAATCCGACTGTTTAGGCTAAAACAAAG
CATCGCGAAGGCCCGCGGCGGAGTGTTGACGATGTGATTTCTGCCAGTGCTCTGAATGTCAAAGTGAAGAAATTC
AATGAAGCGCGGGTAAACGGCAGGGGCCAATGTGAGCTCTCTTAAAAGGTAGCCAAATGCCTCGTCATCTAATTA
GTGACGCGCATGAATGGATGAGTCAGAATTCCCACTGTCCCTACCGTCAGCGAAATACAGCCAAGGAACGGGGC
GGCAGGAATCAGCGAGAAAGAAGACCCTGTTGAGCCTTGACTCTAGTCTGGCGCTGTGAAGGGCACATGAGAGT
GTAGGATAAGTGGGAGGCCCGCTGCGCGTCGTGGGTCACCCGCCAGCGCCGCGGCCGACGCCGGTGAGCCACTA
CTCTGATCGTTTTGCACCGGGTGAGGCGGGGAGGCAGGCCCGGGAGGCTCTCGCTTCTGGCGTAGCGTCCGCTCA
GCGCGCGCCGGGCGCGACCGCTCGGGGACAGCGTCAGGTGGGGAGTTTGACTGGGGCGGTACACCTGTCAAAG
CGTGCGCCCAGGTGTCTAAAGGCAGCTCAGGGAGGCCAGAAACCTCCGTGGAGCAGAAGAATAAACTCGCTTGA
TCTTGTTTCAGTGCGAATACAGACACGTGGCGGACCTGCACGATCCTTCCGACTTTCGGGTTTGAAGCCAGGAGGT
GTCAGAAAGTTACCACAGGGATAGCGGCTTGGCGGCCAAGCACGTTCGCCAGCGACGTCGCTTTTTGATCCTTCG
ATGTCGGCTCTTCCTATCATTGTGAAGCAGAAATTCACCAAAGCGTTGGATTGTTCACCCACTAATAGGGGAGCGT
GAGCTGGGTTTAGACCGTCGTGAGACAGGTTAGTTTACCCTACTGATGATGTGTCGTTGCGCTAGTAATCCTGCTC
AATACGAGAGGAACCGCCCAGGTTCAGATTTGGTACGTGCTTGGCTGAGCCACTGGGCTACCATCTGTGGGATTA
TGACTGAGACGCCTCTAAGTCAGAATCCACGCGGAGCGATACCGCAGCGCCGGGCGCCTCAGTGGGCTGCGATG
CCCGGCCGGCTCAGCTGGCGGCCGGTGCGGAAGCGCCCGTCCGTGGTCGGGAACGGAGCGCGGCCGGAAGCGG
CGCCGCCTCTCACCCGCCCAGCGTACCGCCGTTCGTGGGGAACCGGTGCTAAATCGACCGTGAAGCGACCTGATT
CTGGGTCGGGGTTTCGTACGTAGCCCAGAGCAGCTCCTCGCTACGATCTATTGAGTCAGCCCTCGACACAAGCTTT
GTGGCGGCCGGCGCAGAGCCCTTGACGGAGGAGGCGCGGCTGCTGCGGCCGGCACAGCGGCGGCTGCCCCGCC
CCCTTTCCGCTGCCCCCGGTCTCTCGGTCTCGGTCTCGTTCTCGCTTTGCCGCGGGAGGCCCGACGCGTGCGGGTC
AGGCCCTCCGTGTGCGCGCGCGCGGGGGCCGGGCACAGGAGACCAGCTAGCTGCGTTGCGCTTCGATCTCATTC
GGGGGTTCCGTGCGCCCGTCGCCTGGCCCGTAGCGGAAAGAGTGTCTTGACTGATCATGTGGTGACGTGCAGGC
CCCCACTGGCCGGTTTTCCTGCATCCATCCACCTTCCCGCAGGCGCGGGTGGGCCTGGGCGAACATCCACCCCGCT
CGATCGGCGGGTGCAGTTCCGTCCCCTGCGGGTAGGCGTGCGTCCATGCAGGGGTTGACCTGTCGGCGGTGCGTT
TCCCGTCCGTGCGCGGATAGGCGTGTCAACCGCCTTGAGGCTGACCTGTCGGCCGGTGGTTTCCCCGTCCGTGCC
GCGGGTAGGCGATGTCGGCCCGTAAGCGGGGTTGACCTGTCGGCAGTGCCGTTCAGTCCGTGCCGCGGGTAGGC
GGTGTCGGCCGTAGCCGCGCGTCACGCAGCTGTGTCAAGCGCTAAGGGTGCGACCAGAACGACCAACCGCCCCC
GACCCGACCGACCGCTTCACTTGCGTTGACCCACCATCTTTTTATTACTTTTTTATTTATTTATTTATTTTATTTTACC
GCGACATTTCACGATCTATCTGTACTTTTCTAACTTCTTCTCTTCTACTTTTCTTTGACTTCTGCCTTGCCTTGCCTTGC
CTTGCCTTCCCTTGCCTTGCCTTCCCTTCTCTTCTCTTCTCTTTCTCCTCGTCGCCCTTGGCGTGGTAGAGCCCGGCCC

GGCCTCTGTCACGAAGGGGGTCGCTGCGTGGCCGGGGAGGGATGGGGGAGTCCACGACAGGTTGCGGGTATGC
GGGTAGACCTGGCTGCCGGCCGGCGCCAGATCTTCCCGGCCGGCGTCCATCCCAGTCCACCGCACGAGCGGGAAC
CCGACCCGGTATTACCCGGCGGCGGCCCGCCTCCGCCCTGCCACCGGCCCTCGCCCACAACCCCACCCTCTCCCGG
ACGCATCTCGCCCGACCGGCCCGCTGACCGTGTTTTTATTTGTTTGTTTGTCTTTGTTTTTGTTTTTTTTTTTGGGTCG
GTCGGTGGTCACTCGCTCGGTTACTTGTTTGGACCCGATTGGCCCGTTTGATTTTCTTTTCTTTCCTTTTCCGCGGGG
GTGCTCCTGGCAGTTTCGGCCCAATGGCGCTCGACTCGGCGACCCGGAGCCCGCCGCGCCTCGCCACCCCAGCCC
CACCGCCACCACCGCCCACCACCTCCTCCTCCGCCCGAATCACGTACCGTTCCACCTCATCTGCCGGCTGGGAGAG
GCTAGGCGGAAACCCCGCAGGGGACGCAGCCGCCCTCCTCAGCCCACCTCTCGCCGCTGCCGTCTTCCCGCCCGCT
CTTCGCCATCCCCGCCACGCGGCTTACTGCCCCGCGATCCGGATATCCGGCCCTGACGGACCTTGGTGGCGGGCCC
GGCGCACGCGCCGCGCTCTTCCTGCAGAGCCGGGGAGAGAGGGAGCAGGCAGCTTTCAAAATCACAAAAAGGG
AAAGCAAAGAGAGCCCACGAGAAAAACAAGCAAACAAACAAACAAGCGAAATAGTAAAACCGAGAGGGCGGGG
GCACGGACAAACAATGAGCGGACCAAAGTGCACGAGCAGACGGGCGAACAAACAAACACGCACTTGAACTTAAA
GGAGCAAAAACCGCGGTGGCGGTGGCGGGATGACCAGCTTCAAGCAAGGCCGAACCAGAAAGCCAGTGCGCTT
GACCGAGCGAACAGCGGCCCCGGCTCATACGTGCACCCTGGTCGTTCGCGGCTGCGGCCAGGTAGAGCGTCTCA
GTGGCGCCTCCGGGCCTCCCGGCACGGTCTACCCGGCTCCGAGCGGGCCGTGGTGCTGTGGCCCGGCAGAATCG
CTGACTGGGCACCGACTGGAGTCGGCCGCGGTCTACCCGGCTCGGCGGGCCGTGGTGCTCGGCGGCTGCTAGAG
GTAAACTGCCTATGCGGCTCGACTAGTCGGCACGGTCTACCCGGCTCGAGCGGGCCGTGGTGCTCGGCGGCGGCT
GCTAGGGAATTCGCTGCCTATGCGGCTTCGACTGGGTCGGCACGGTCTGCCCTGCTCCGTGGAGTGCGAGGGCTC
GCGGCGGTCGCTAGGTGTCGCTGCACGTCTGCCAGCCCGTCCGTCGCTGACCTCACAGCGTCCCGCGGCAGAGCA
GGGTAGATGCCCGGCGTGGAAGCCGCTTTACCGCTCCAGCGACCCCTGGCGCCACTGCGCTGAGAGCTTGATTTT
CGGGCCGAGGCAGGCCGTGCCGACCAACTCGAGCGGGCGTGCAGCGACCGCTGGCGGAGGCTCGTGTACCGGC
ACAGTCCAACCCCGCTCAGCGGGTTCCGCTCGGCTCGGACGCTCTGGGGCGGAGTCGGGGCGTCTGAGCCGCGG
CGGCTCGCGGTGCGCCGGCACGGTCCCAACCGCTCGAGCCCGGGCTCGGCTCGGCTCGGGCGTTCGCGGCAGCG
TCAAGGAGTCTCCAGCCGCGGGAGGCTCGGGTGCGCCGGCACGGTCCAGCCGCTCTCGGCGGGGCTCACCGGCT
TTCGGGCGTTCAGCGGCGTCCGGGGGTCTCAGCCGCGGAGGCTCAGGTGCGCCAACGCGAT

>14.91.732649fb-8ab2-4fe0-93a7-9a06d29821ab protocol_group_id=2020-02-09_1st ch=408
barcode=BC02 read=3179 start_time=2020-02-09T17:03:29Z flow_cell_id=FAK79217
runid=88172aec3f27372c4684111da770399a9fd1fb40 sample_id=2020-02-09_1st

TCAGGTAGAGGGTAAACTACATCCAAAACTGGAAAATGAACACTGGGGGTGCGAGCCCAGAGCAAGAGGAAAA
ATACTTTTTCTTAAAAGTATTTTAAACTGATTTTTCATTCTTGCTGACATCCAATTCCTTCCCTCAGAAAGCATTACTC
TATTATAAGGCTGAAAAACAAAGTTTTCCCAGCCAATGCAAGAGTGGTTGTTGTGGGACATCAGGAGGTCATCTG
GTTAATGTCCTATTAAACCTTCATCAGTTTTACCAGGACCAAGTACAGTTTATAGACTTTTGTATAGATGCAATCATA
TCTGCTGTACATCTTATATCTATCTACACATATAGTATATATATCTATAAAATGTAATATACAGTATCTATATGCATA
TGCACTTACATATTTCCTTACAGGTAGACTAGATATTCTATGTATTTGGATGCTTAAAATATATATTAAAATATATAT
AAGTTATAGACAAATTTATAACTTATTCTGTGTAGATACATAGAGATGTTGTAGAGAAAAATGTAACATACAGATA
ATATCATGGGTCAAGCCTCATCCATTCTGTAGTCATAAGGAAGAGACTGTGACATTTGTTAAAAACCCTAGAGCTG
CACCTGCAGTCATAGTATTGGGTAGCAAAGGAAGCTATGGTACAATCAGCCACACAGAGCTAGATCTCATCCTTAC
TGCCTTGCTGCCTTAATAGATCACTATTTTTATTTTTATTCCATTTATTTTATCTTACTCTCCTTGCAACCTTGAAAGTC
ACAGAAATCTTTAGTCATGTGGGCGATGTTGCAAATGTGATCTTTGTAAAAGCATAAAATCTTAAGAAAACAGAAG
TAGGTTCAATTCCAGCATTTTGTTAAGATAACATGTTTTGCTTCATGGAGGAAAGTCCTTTAGTACCTAACAGCCTG
GTAACATTTATAAGAATGTGAGAACGCTGGCATCTGTAACAAAGGTGACAGTAACTGTCTGTTGACACAAGTTTGT
GCATTAGTAATTTTGTTTGTTTGTTTTATACACGGGAACCAGTTCGGGCATAATTCTTATCTGAGCAGTAACTGTTC
GCAGGGATATGCAGATTACTTACGGGCCTCTGTAAATCTGACTATAGCTGTGTCAGTTGAGAATCTAACCATCCTT
GCCCTGGCCTGAGGGTGAATCTACTGAAACACTCGATTTTTCTGCCTCCCTTGCCTAAATATGAATAAGAGCCTTCT
GAGAATCAAACCAGATGTGCCTTCTATGAATTGATACAAATCATACCAGATGCAGGTAGGTTTTTCGGTAGTACCT
AGATACGCTTAACACTAATACTGGTATCAGTTGTTGTTTTCTTGTTGTCAGTTGTTGGTATAAGGACAATAAGTAAA
GCCACTTATTGCATTGTTTTAGGTGGAGTTGCTTTGCAAACTGCAGCTTGAAAACAACATTAGGTGCTAAAGTAGT

TTACTGCAGTAGTAACAAATTAAAAGCAGCACACTGTTACATCAGCGATGTACAGTTTCTGCCACTTGATTTATGAG
GTCAGGGTAAAAGAATTTATCACCTGGCATTATCTCTACCCAGATTTAGCTGCTCTTAATAAATCAATGCACAAAAA
TATATATACAGAAGTAGAGAAAGGTCTTTCAAAGACATGGGCTGTTTACAAAGGAGTGTCTCTCTGTGAGACACCT
TAATAAAACAACAGCCTCCAGTGGCTAAATCTCACAGCATAGTGAGCAATCTTTATGGTAGTGGCTTTTTACTCCAG
ACAAATATTTTAAATTTGTTGTCAAATGGCAAGGACTGTCATGTCACCTCCAGCAGTAAAGGGAACATATTAAGCT
TTGGACTGCCTGTATCTTTGTTCCCCTTTCTGTCTCCTGGTAGCACAAAATTAATGACAGACTAGAGACCCTCTGCT
GAAGTTCCCAGCCCAACATGTTAACCATTAACTCAAAAAGTCTCCTTTCAAAATAAGCACTGACAAAGAACAGAGA
GAACTACATTGGTTCTGAAAAATATCATAGGAGAAGAGAAAGGGAAGCTAGTTCTTGATTTATGGTGAGGCTTCA
TCTTTTTCTTTTTTTTCTTTTTCTTTTCTTTCTTTTTTTCTTTTTCTTTTCTGAATTCTGTAATGTAGGACTGTTGCCAAGT
CTCACCTCAAGAGATGACTGAACTCCAATGACAGATAAATACTCTCTACTGTATGCTGAGCGCGTTCCAATACTTCC
TGGATTTGAAAAAGGGTTACAAACAAAGGGGTTATTATAGTAGCATTTGCCTGAAGAGACAAATAAGTGTGTTCA
GGAGACTACAGTGACATCAGCATAATAGCTTTACTCTTATTGTAATCTTTGACATAGAATGATCTTCAATTTTCACTA
TGCATGTAAATGCTCCAAGTCCCAAAAGTTCTAATTCACAGTCTTATGATAGATAATTAATACCTATTTACTGCAGA
GACAGTAAAATTCATTGAGGAAAACTATCAAGTAATTCACATAATAAATAAATAAATAAATAGAAGAATTTCAAAT
ATTTTATTAAAATCCAGTAAGAATTTTTTCAACATTACTTCATTTATAGTTAAACTAAGCACAAATTAGTATCATAAA
AGAAAGTTGCAAATACATTGTGAAAAGTCAGCAAAAATCTTTAATTTTTGAACGGTGTCTCTCAAAAAGAAAACTT
TACTTGGAGTCATATAAAACACTGGTCTGGCTGGGGGCAGCTTAAGGCTGCTTTGTGCTTTTTGGAGTTGTATTCT
GCTGAACGTTGGCAAACACCTGAGCTTTGCTACTGGCCAGTCTATACCTAATTAAAAGTCTTAATCTCTGAACAAGC
TCAAGAGTTTGGAAAAAGACAGCTTTAGCTTTAGCTCATTTAATTACAAATCATACAGTCATAATGGAGTACATCCA
GGTGCAGTACCCTCCAGCTGTCTGTCACTATACAATCATTCATGTGAAAATAATAATTTGAATATATTGCAATTAAA
AACTATTCAAAATGTTAGACTTTGTTGTAGAAGTTTTTGCTTTCCATTTCCTCATTTTAAGTAGTAATAAAGCAATTA
TAAGTGTCACTTTAAACTTTGCATTTTAGAGACTTTGGAACATAAATGATACAGTACACCAAAGTAACGGTTTGTTG
GAAATTTATAAATTCATTGTCAAATGATGAGGACTGTCATGTCACCATAGAAATTAGACTTCATACACTTCCCAATT
CATCTACTAGGGGCCTTTGCTCATGCTTAATTTTGCTACAAAACAAACACATTTCATAACATAAAAACAAACTATTTC
TCATAGTAACCAGAGGAAAACATACAGTTGTTATTTTGTATGCAGGGTAGTTTTTCTCCCATATGTGCTGATGGGA
TAAAAAGTTTCACAGGCACTTGGCAGTCCTCATCCATTTGACAATGAATTTATAAATTTCCAACAAGCCGTTTACTTT
GGTGTACTGTATCATTTATGTTCCAAAGTCTCTAAAATGCCCAGTTTAAAGTGACACTTATAATTTGCTTTGTACTAC
CCAAAATGAAATGAAGCAAGCTCCCAGCCTGGTCACTGCATTTTTAGGCTGTGGACACAGTAAAACCAAGTATTAT
TTTCACATGGTATTGCTAGTGATGAACGGCTACGCACAGATGTGCTCCGGCTGTATGATATGAGTAGAAAATGACT
AGCAAAGCTGTCTTTCCAACTCTTCAGTTATTCGAAGATGGGCACAGTAGGCATGAACTGACCAGTAGCCAGGTGT
TACAACGTTCAGAATTTGGCTCGGCAAAAACACACAAATGGCGAGCCCATATGGGCCTTAAGCGGACACGAGAG
GCACTAAGTTCAAAAGGTGGGCACAGAGCTTTCTGAATGTGACCACGCTTGATACTGGCGTGATTTGGCGCTGGC
TTGTGTTGAAAACACACTGGATTTTTAATAAAGTATTTAATTCCTTTCTGGTATTTGACTATTTATTATGTGGTGTAC
CGATATTTTCCAGTAATTTTACTGTCTCTGTGCCCGGTAAGCTGAGGCCGATGATGTCATCATGGGCTGTGAATTA
GGCTTTTGAGACTAGTATGTAGTGAAAAATTGGTCGACATGATGGTACAATAGCAAAGCTGGTATGTTGATAAAA
GTCGCACCCAGCAGTCTCCGCATACTTATTTGTCTGTATAACACTGTGAAATCAAAATGTAATTTAATACATACAGA
AATGAAGTATTTATCTGATCGTCTGGTGAACGCTGCCGGTGATACGGGGCTTTAGAAAGAAAGAAAGAAAGAAA
GAGAAAAGCGAGCACTTCGCCGTAAAATCAGGGCTAGCTTTCATATCTTCTCTAACCACTGCAGGGAACAATGTAT
ACGATTTACTGTGGTAACGACACGAAGGAGGCTTTGGTTAATGGTTAACATGTTGTCTGAACTCGGCTGGAAGTCT
TCGATCGTGAACACTGACCCAGCGCGAAACGAAAGAAACAAGATACCGAACGTCAAAGCGGCTATGTTCCTCACA
CGTGAAGGTGTTATATTCTTGCACCATTGTTAACAGAGGCTAAAACCATTTGATCTGAATTGAAACCTACCAGGAG
TAACTCGCGAACTCTTTGTTTAGGCGCGAGGCTGTAAGATTGATGCCACGGAAGTTTTGCTCGCCAACCAAACGGC
CATGTCTTTGGAGACTGCTGCTCACTCTATTGTGCGTGGCTGGGAAGCAGGCGTGTCTGGGTGAAATGCCGGGTG
GAAATTCTTTGCTGACCTCGCCAAATCGAAGTGGCAGGGCATGCGTCGCCGTTGCGGTGATGCCGTTTTTGGTACG
TTGCTGCTGCGGTGCAAATATTACGCGCCTTAACGTTGCGCCACTGCTTTGCGGTTCTGAAAGCAACCTGCCCAAA
ACGGCGCGGTAAAGTGGCTTTATTCGACCCGATAAACGCCCGGCTGACGGCAGAAAGCGGCTGTTGCCATGTGTG
TTGCTACGTATCTAGGCTACTGCAAAACCCGCCTGCATCTGTTATGATTTAATCAGTATAGAAGGTTGTCAACATCT
GGTCAGAAGGCACTTATTCATGGCCCGAGCGAGGGAACGAAAAAAGAATCTTTAACCCCCAGGGCAGAGAATAG
ACACTCAGCTTTGACTTGCGGCTACTCGTCGGAGCTTACGGAGAAAGTCTGCTGTCTGCATATCTGAGTGGCCGGT
TACTGTCGAATAAGGAAGCGTAAATCGGTTCCACAATAAAACAGAGCAGGCAAGGTACAATGCGCAAACTTGTGG

GGTTGCTGTCACCTTGTTAGATGCCATATTCTTATGGAGTTGTGACGACACTGGAGTTTGATGGGTACTAAAAACC
ACATGGTAAGAAGCAACATCACTGACAGATGGAGTCCCCATTTCTTTTAATTATCTATAGCACAAAAGATGCATTA
CAGCATCGCCCTTGACAGATTTCTCAGGGTGTGGTAAGATAAATAAATGGAATAAGTAAAATAGTGGTCATTAGT
ATGATGTGTAACATTCAAACATGGCCCCATACCCACCTATGACTTGCAGAACTCTAGGAGTTTTGAAATAGCCACA
TCTTCCTTGGTATTTACAGAATGGATGAGACTGACATATTATCTGCATGATGACCATGCTCTACATATACCATGCTA
TCACAGAATAAGTTGCATTAATCTCTTTACTGTATGTTACAATATATTTTTAAGCATGGAGGCATCTGTATAATAAT
GAGTGCTGTACGCCTTTATATACATAATTATATACGCATATGTGGTGAGATAAAGATGTACCGGTTATAGATATAG
GTGAGAAACAGCTCAAAGAGGTCACGCAAACATTGCTTGGTCCTGAAGCTTGTGAAGACGATGGGGGCGTGAAC
AGGCCAGGTCACTTGGCTGGCATCAGCTCATTCTGATGTGTTTGTTTTCATACACTAATGGAAGTAATGCGCGAGG
AAACGGATGTCAGCAGAGTAAAATCGGGCCTAAATATGCTAGAAAAAGTATTTTCCTGCGCTCACCCAGTATATTT
CCAGTTTTGATTTACCTCTTACCTGCTATCATTGTTCTGTTTAACAGCGGCAACTGCGCGGC

>12.332.209704ed-d353-4764-963e-4f4dd1161d43 protocol_group_id=2020-02-09_1st ch=242 barcode=BC02 read=12800 start_time=2020-02-09T16:50:39Z flow_cell_id=FAK79217 runid=88172aec3f27372c4684111da770399a9fd1fb40 sample_id=2020-02-09_1st

TCATTCCTGAGACCAACAAGTTATAGGGATGGTTACAGTACATGGTTCACTTTACAGCCTTCTGAACTTTGTGTCTT
AAAAGGACGAGAAGAAACTGCTGAAGCTTCTGAGCCGAGAAAAATGTGGGTGATTCGGTCCTTAATCATCACATA
ATGCCCCCATGGTTTCAACACTGCAAATAGGAGGAAGGTAATAAATTTGCTGTTGCGGAAAGGAAGCCAAACATC
CTGGGATGACTGCTTCAATAAAAATCAGAGCAGTGGCTGAGCTTAGGAAAACTCATATGAAAGTATTTACTGAAA
CCTGATGGTTAGATGAAAAGAGCTTTAACATTCAAGCAGGAAGCTCCATGAGATTTAATAAACAAGCAGAAGAAG
AGCAAGAAGAAGCAAGAAGATACAGATCAATAATCCCTTCCAGAAGAAAATGACTACATTCCTTGATTTAGAGGA
ACACATGAAAAGGGAGACCCCAAAGCTGAAAAAGCAGTCTTAATACAAAACAGTTCCTAATAAAGGTTGCTGATG
AAAACAACCAGACTATTAAGTGTACCCATTGCTGAGATTAATATAGACAAAACCTACACTAATATTGTTTATATGTT
CACATATAAGGATTTATTTCTAGAAAACAAACCAACAAGAGCATGCTTGTTGATTCATCCAATATTGTTGCTCTTCT
ATTAATTCCTCTACTGTACCACTGGAGTAAGCAACAATCACAGGCAAAGCAAAAAGAGAAAAGATCTTTGAAGAA
ACATTGTGATCTGACAGTAGAAATGATGCTCCATCTCTAGAGGATGGTCAAGGTTTCCAGCTGGAAAATCAAGTGT
TAAAGAGTACAGAGAAAGGAAGAAGGAAAAGAGAAGGAAACCAGCAGATCTATTAAAAGCTGAGAAAATATGC
AATGGTTTCCTCCCTAAACCAAAAATAGCACAATTATCATATGCAAAGGCTAGGTCAGGCACCCATTGGCCTCAGA
AGGTTTTACACAGCATGTTGCATCACGCGTTTACAGATCTCCTTAGGATGTTAATGTGGTTCAGTGGGCCACAAAA
GGGAGTTTGCTTCTCAGAGAACTGAAGACCATTTTATCCAGCTTCTGTGTTTTGTGAGGAAGAATTTTGTGGAAAT
GGCTTGAGAAGTCAGAATACAGAGACACATGATGTTAAAATTGCCTCATTTAGATAGCATTAACACTACTGTAACA
AACATTTCTATGTTATGACAAGCCCTAGATTGAGTTTGAACTATTCTTCTGTTTCCTGGCAAGAAGTGACTGCCATA
ACATGATTGGGAATGCAAGAGGATTTCAATAAAGCATAATCCTGTTCAATCTTCCATACACAAGTCCAGAGCTGTA
AGAACTTCACTAACTATAAGACAGAAATAAAACCACATTGTTTCAATCTCACAGCTCCTGCTTCTGCATCACCACCC
CAAATGAAGTCATAGCCTTCCAGTTCTCCACATCCCTAAGGTTACATATCTAACCTGGAGGCAGTTGTTCTCTTCAC
CAGGCCAGCCCACAAAACAGAGCTGCAGTGTGCTCAGAGCAATATCATTTGGGACCGTATGATGATCCTTCCACA
GAGTATAGCACAGTCGAGCATAAAAAAAGGATACATTCCAAAGACAACTATTCCACATTGGAATACCAGTGCTTTA
GAATTATATTCTATGTAGGAAAAAAAAACAAACCATGTACGATTTTGTTTGGATACATGTTTTACTGCTGTTGCTTT
CAAAAACAAAAAAGCCCATTTTGCGATTACCTTCCTACATAATCAGAAAAAATCTTGCCAGTACAAAGCTAAAATAT
TCTGTTGCTTAGTAGTTCTCATCTCTTAAAACAAAACGGCACAAAGGAACAGCACAGCCTCAGAAACAGAAGCAGT
GCATTATTTCATGCTTTCAAAACAATAATCTCATCTTTACCAGCACTGAAGCTTTGAAGCTTATACATCTGTGCAACT
GAATGTTTTTATGGCTCTTAAGGAATCATTAGTCACTCTTACATCCCTTCTTTTCTTTCACGATAACAAAATGAACGT
GTCAGCTATACAGGAGAATTCTCAGACCACGATGCTATATGTTTAGTTCCACAGAAGCACCAGCAGATCTCACGTG
CCTCATGGTCATTACATGCTCAGATGCTGTGGGTACAGTTAACACCTGGAAGCCATAACACAATTTACACGCTTAC
AGAAATTCATAGTAATTAAACGTCAGGTAATTAATCCCTTAACACTGTGACCACTTTCTTTTTCTCCCCTTGAAATCC
CTGTCACCAAGGACAGTGAAAACAGAAAGCGCGCTGGTCCTAGAGAAACTTCCGCCAGAATAAACAGCCATTGCT
GGGGACTTCAGTCTGTGTAGCGCTGTGGTCTACAGACATTTGTAGAATCATGGTGCAGAACAAGGAGGCCAAAGA
TATTAGCAATTCTGCTTCACCATCCACTTTCATTTCTCTGCTGTTGTATGGCATGGCAATTCTGAAAGCCTCGTTTCC
TCAGAACCTCAGCACTAAGCCTCAGAATGTTGGTACCTTCTGGCAGGTTTACTTTGCCTTGGAGCATCAAATGACA
GAATGGTTTTGTGTAGGAAAGGACCTTTCAGATCATACACAGCTCCAATCCTGTAAATAGGACACACTTGAAACAA

CTTATGGCAAATTCTGCTGATTCCAAATCTTTCAAACAACATTGATATTCATCTTAGTTGTCTAAAGCACTCTGATTT
GAGGCAACAAGGCTACACGCTAACTGGATGAGTAGTCTGCCTTTTCCAGCACCTTCTTACGCACTGTATGCTGGAG
CCTATTTAAACAACAAACCAGAAGGAAAAAAACAACAGAATTGAATCAGCATCAGAACACTCTATCCAGATATCTA
TTATTTTATGGAACAGAGGTAGGGAAGGGAAGTTTAGGCAGAGATCAATGGGTTAATGCTGTTAAGCTGCGCACC
TAAACTGAAGCGAATTTCTGTTCATACAGACAGACGTACCCGAATTTACAAGTGTCCCAGAGTAATATATTGCAAA
TTGGCTTCATAAAGCATCTGAACAGGCAGAAAGAAAAACTCGTGCTACTCTTCCATAGGGCAGAAAAATGTGCCC
CAAAACATTCATGAACTCTCCATACAACACAGAAGTTTAAGGGGTGCGTGTGCAATTGCTGGGTTAATCAGCAGA
ACATATGGTGGACAGCTGATCTAATAATGCTTCAGATAGATATCCATGATGCTAAATATACAGAAACCATTACCCC
CCTTCTTCCCCACCATTTCCCAAGATTTCCTGCTTTAGCTGGAACAGAAGTCAGAGCAAACACAAGCTGCATCCTGT
GACTGATGGAACAAGAGATATTGATATGAGGAGGATGTAAAAACCCAAATCCATCAGACCCAAGGTTTATTTGTG
AAGTTGAAGGCATCAATGCTCTGCAGCCTTTGGAGCATGCAGCCCATTCAGCTGATGCAGGAAAGAAAACATCCC
AGAGGAGTTGCACTAGAACTCTCCCTCAGCAGAAGGAAGCTGAACCTGCTGCTCTGAACTGTAGTGAATTAATGTT
CACCTATGTAACTCTAATGCAAAAAAGTACACGTGACTGGAACAGCTTCCCTCCAGCCTGAACAACCCCATTCCCA
GCAGCCCACAAAAGCCATCTTTTTCTGAAAACATAACATAGAGAAAAGATAGGACTATACTTATGCAAGGTATTTT
ATTTGATTTATAGATGATTGCATCTCTTGCTACACAAGCCACCGTGAGTGATAACAAATTACAAAAGGGATGCATG
AAATTACAGGTACGTTGTCAGAGATGCCAAGCGAAGATGCAAAGGTACATCATTGCAACAGCCAGTGAAATGACT
CTTTTTGTTTGAAACGTTCCTACGGTGTACACAAGTTCCTGTCTGGTTGTTGGTTTGAGTTTCCAAGGAGGAAGGA
AAGAAACCACTCTCTTCATGCTCTAACAGAGACTCAGATAAGCAACAGTAGTGCTTAAAGAGTGCTGGTACACCTC
AGAAGAGTTCACTGCAGCTTTCCTAGGGTTATCCCAAGCACTGAACTGCTGCTGCTGCTCATCCTGTGCATCAGGC
AGTGGTCTGGGTCCTACCCAGAAGATGATCCCGCTATTCCTCTTCACCTTCCTTGCTTGCCAAAGAGCCACATGCAC
CAGAGTCAAGGTGAAGCCCAGCTGAGAGGCAGTGTTTGCTCAGCACTAGCACTGCATTGGTGCACTGACGCTTCT
GGACCAAACACCTATGGCCTGCAGGTATACTATGACGGGAACCAGTGCTAAGTGAACGGTTTGGGGTAGACTAG
ATGACCTTAGTGGTGCTTTCCAACATTAATGCTTCTAATTCAACAACCGTGTACTGACAGGCACTCACTGCAACTCC
TATCCACATGGATCTCAGCAAAACGAAGCTGAGCACAAAGGCAGCCAGAAATGTCCATACATGCTCTCGCACATCT
TGCAGGATTGACAGCAAGCAGTATTTCTAGAGAGGTGACATCCTTCGGTCCCTGTCACAGGCCTCCTGGAGACCA
GAAGTCTCCTCATCTACTCTGAAAGATGGCTTAGAACCCCAAGGGAACTGAAACCCACCTGATAAATAAGGAAACT
AAAAACACACCCATGGGGTATTAAGAGTTAAAGACTGAGAGAACAAAGTAGATTTAGTTTTCTTTTTCCCAATAGG
ACCTGTTTACAATAGTAAAAAGTCTCACATGCAAAAATGCAAACATCACTATAATCTAAGAAAGCACACAGAAATT
AAAGCAAGCAACTAAGCAGTCATTTATTGAATATTAATGAGCCTAAAAAGCTAAATCCAGCTGCAAAAAGTTCTTT
AATATAAAAAAAAAAAGCAAAAGAAGATTGTTTTCAAGCTGATTTATTATTTTAAACTGAATAGCTTTGCATGCGAA
ATGCAAGAAGACCAACTTCATCATCTTGCCCTAGAAAGCTTCAAAAATGGGCCAAGCAAAGATCTTTAAAAACATC
GGATATCTGGATGGAGTCAGCATTTAGCTGATACACTGAGCTGGGGATGTCACCGGACAGCAGCTCGTTCCAAAT
CTCCACATGCATTTTCTATATGCAGGAATCAAAAGAATGCATATATTTCTTCCTTCCTGTCAGGAAGCCCCAGGAGC
TTTCCACCTGACAAGTGAGCAATGCAATTCATACGCCACAGGAAGTTTAGGACAAAATCGCGAAGACAGGCTTTC
ACAGCATCCTCAAAGCACTGGCAGCTCGGGATGGTTTGGTGAGTGTTGGCCTCACTTCAAACGGAATGCTTTGAA
ATCCACCAACTCAGGGGAGGAACAGAAAGCTTTATGTCAGCCAGGATCCACTTAATTCTTAATGTGTTGGTAACCT
GCAGAATCTACACCACCTCTGGCTGCAGACAGTAAAGAGACAACAGTACTTGCTTGTATTCTGCACGTCATCTCAC
CCTCATGTATGTGTCTGGGTGAGAGGTCACTTATGAGAACCACCAGCTCCTCTCCACGTTTTAAAAAAGAGAGAAA
TTGAGGACTTACTCTGGTACAGTCCACAATCCAGCTGTCTACAGTGCTTGTCATGGTTTAATAATTTTGTAATTTTTG
CTATCAGTATTCCACATCATAACATCATATTAAGCATGAATAATTTTACTGAATCTGCAAGCTTACAGAAAGAAGAC
TACATCTCCCAGGGAACAACCACGATGGGTCACGGACCTGGACTCTATATTAACTCAGTCGCAGAACAGACTGAC
GTCTTTCGAGTTCTGGCGTTCAGGGGAGTGTGTGGGAGCCTTCAGCCGTTCGCCTAGATTCACAGTAGGCCTCTCG
ATTCACGAGGACTCATTCTCTCTCCTATTTTTGCTTGATTCGTTAGCTCAATTTTCAATTATATTGTATTATGTCATGT
TATCCTGTATTTCGATATAGTATTTAGTAAATAAGTGTGCCTCCTTAGATCGTTGCCGCTGTTTTCTTTTCCTAATTAA
CTTTTTTCCTTTTCGGCCTAGAGGCCTCGAGTCATGGCCCCTGTCACGGACACAGGTGGATTTTTAGTTCAAGCCGT
GACAGATTTTGATATGAGAATGCGGGCAAATTATGAGTTGCTGTTTTAATGTGCTTCTTCTGTTGCTCTTTTGGCTT
TTAACAGGCTACGAGTTTTTCCCTGTTAAAGGAGCTATCTGCGTGTTTGCGATTCTGCACCTGCGATGACCTTGAAA
GTCCAGAGCGGACTGCCAATACTCGGTATATTTTATAAATACTGCTGAGCTTTGTTTTCTTTTACTTTTTCTCTATTG
GAAAAAAAAAAATGTTTTCTCTACTAAATTTGTTTTTGCAGGGACTGTGTGCCATTGGATTTCCCTTTTGAGACGCTC
CGAACATATTGGTCATGTTTAAGCTAGCCACTTTTTCTTATGAGCTATACAATTGTATAATGGATTGAAAGCATGGT

TAGCAATGCTCTCACTTCGTATCTGTATACAGCTCAGAAGGAGCAACTAATGCAACTAATTATACAGAAATGGTGA
TTTTTAAGCGTGCTCCTCTTTCTTTCTATGAATCTTTTTTTATGCAGTTTTGAATGATCAGTGGGCCACACTCTCAATC
TCCCTGATTACTGTTGATTGTGTAATAATCTCACTCAGTATCTGGATTTAACATCCGGAGAAGTCTTCTCTAGAACC
AGAAAATGCTGAGTGGCGGGGAGTGTGGAGAGGCTTTGAAGAATTCTCAAGGACAGACATCTTCAGTGTTATGG
AGCTTCACTGGGAACACTTGAAGAATCCTGAGAGTTTATCAGTTGCATATTTGAGGCAGAATGTTGTGGCTCAGGC
AGATCTCAGGAGGCACAAATGATTTTGGGCCTAGCTAATGCTTATCGGGCCCTTAGTCAATACCATTCCTGAGAGG
AAGTTTTGAAGCTGAGAGAGAGTCTCAGGGCCGAGCGGAATCTTAGGCTGAGGCAGAAAAACTTTGAGAGGAAG
GTAGAGAGAGAAAAAAGCTTTGAAGCTGAAAGGAGAAAGATCTCTGTACCAACGAGACTCAATCTGGTACGAGA
AAGAGAGTCTTAGAGCTGACTTAGAGAAAACTTTGGGATGAGAGGTAGAGAAAAGTTTTTGGAAGGCTGAGAGA
AAAAGATCTCCTGTACCAGCGAGACTCAATCTGGTACGAGAAAGAATCTTAAGAGCTGGCCTTAAGAAAAACTTT
GGGATGAAGAAAAGCTCCTGCAATCCCAACTCAGCACTCTCAGTCCGAGCACAGCGCCACTCCGGTCAGGCACGA
CACTCTCAGTCGAGCACGGCGCCCTCAGTCGACACGACACTCTCAGTCTGAGCACAGCGCCCTCCGGTCCGAGTAC
ACTCTCCAGTCGAGCACGGCTGCCCTCCAGTCGAGCACGACACTCTCCAGTCAGAGCACAGCGCCCTCGGTCCAGT
ACAACACTCTCCAGACTGGGCACGATGCCCTCCGGACCGAGCACAATGCCCTCCAGGCCGAGACACAACACTCTC
AGTCCGTATGACGCCGTCCAGTCTGAGTCGGCACCCTCCAGGCCAAGCATGCCTTTCAATCCAGCTGCGACACCCA
CTCCAGTCCCGAGCACAACACTCTTCCAGTCTGTGCATAATACCTCGGTCAGGCATAACGCTCTTCAGACTGAGCAC
AATGCCCTCGGACCGGAGCACAACACTCTTCAGTCCGAGCATAATGCCCTCAGTCGAGCACTTAACGCTCTTCAGA
CTAAGCTGCTCTCCAGACTGAGCACACTGCCCTCCAGGCCGAGCATAACGTCCTCCGGTTCGGGTGTACTCTCAGT
CCAACTACAACAGCCTCCAGACTGAGCGAATTTGCCGTCAGTTGGAGGCAGAACACTCTCCGAGACCAGCTATGC
CCTCCAGAATTGAGTTGAATGTATTTAACCAACTACCAGGGGCACATGTAGTCCAATCTAACCAGCCACCGGGGGC
ACAGGTAGTCCAATTAGGACCAACAAATGCAGGACACAGATACAATGTTAGTGCCTGTAAGAGGGCAGAAAAGA
CAAAACAGGATTTCAACTCAGTTAGAGAAAATGCAGAGGAGTTAGAGAGACGGAGGGAGTTAGGAGGCAGGAC
TCAGGAGACTCAGGGCAGGGAGTCAGAAGGACCAGAAGAAGTTGGAGGAGCAGAGGATGTTAGAGGGAGTAT
AAGAAGATGATAGAGGAGCAGAAGATGATAGGAGCAGAAGAAGTTAGAGGGCAGAAAGTTAAGGAGAGGAAA
AAGAAGGAGGGTTACCTGAATAACACAAGCATCAGGGTCAGGGTCAGAGTCAGAAGAGGAGCCAAAAGCTACTT
TTCTTTACGAAATTTCTCAAGTCCAAACTTGTAGATGGGGCAGCCTCATCAACACAGTTGCCACCATCAACACCTGG
GACTCTTGATAGAGCAAAGGTGAGCAAAGTATTACGATCATTATTGATCGATCTTTTGACTGAGTGAAATTCGAGA
TCTAAGAAAGGATTACACACGTCAGCCAAAAGAAAATATGGTCACTTGGTTGCGCTGCTGGGACAATGGTGCCAG
CAGTGCGTGGCTCGATGGCAGTGGCTCGCCAGCTGGGTAGCATTTCCGAAAGGGACCCACGTGTGGACAGAGGT
ATTGGCACATGCCAAAATCGGTCCTACACCCTCTGGAAGCGGATGTTGATAGCAGTAGAGATAGATTCCCCTTTAA
ACGGGACTTGGAGCCTGAGAAAGAGAAATGGACTGATATTGAGAAAGGTATCCAATTTTGAGAAAATGCTCTGGT
GGAAATGCTATACAGCCTTGAGTTCAATCGTAACGATCCAGACCAATTACATGATCCTGAAGCCCAGTCGCGTTCC
ACGACAAATGTGGCGTGACTTTTACAAAAGCAGCTCCAGAAAAGCATGCCAGTGCACTTGCATTAGTATATGGCA
AAGGGACAGGAAACCCTTTGTGAATATGAACTTAATTGGCGAGCTTCAGGAACTTGAATTTATAAGCGCCACAGA
AGTTAATCTGCACATACTTCAGCCATTGTCAAAATTGTGGGAAGGTTAAACGAAGTGGAGGGAACACAAAATGAT
ATTATTGACAAATTGGCTAATGTGCAG

>10.588. 3e7fe50b-d73b-428f-b838-1a671fa5a4fa protocol_group_id=2020-02-09_1st ch=241 barcode=BC02 read=10939 start_time=2020-02-09T16:38:25Z flow_cell_id=FAK79217 runid=88172aec3f27372c4684111da770399a9fd1fb40 sample_id=2020-02-09_1st

TATCATGGCAGAAAAACTCTTCAGCAATAATTCTTGCACCGTGGTTGCAGGTACGCTGTACAAGAACTGCATTCTC
CAGGCACTTTCCCAGCATAACTAAATCAAACTCAAATGTGCAGAATTCTTAGCTGTTCCTCATAACCTCTTGAGATT
GCTGCACCCAGAGCTCCACAACCTCTAAAGACAAGTCTCTTTACATCAGTATCAACAAATAAAACAAACCTTTACTA
GTACAGGAGCTCTGGAGTTCAGTCAATTGCTTAACATCAGAACACACTAACATCATTTTAAGTACTGCCTCTAGAA
GCTACACTGTTGCCACCAACACTGGCCATCCTTGGTTTCCACATTCTCAGCTGCATACTACTTGCATAGGTATTTACA
GGCAGCTTTTCCAACTCCTTGGAAATACAGGCATACAACTAAACTCCAGTTGCTCACCGCAATGGAGGTATCTAAA
TGAAGTAGTATGATTCACCAAAACACCAGGTCAAACCTGGTTTCAACAGAAACAATTGCCAATGCATCACCCCATA
AACTGCAGAGGCAGAAGTTGCTCACACTGAAGGAGAAGTGGCCCAGGTGAGTTGGGGACAGAACAGAGACAGC

CCAGTAGAACCACATGGACAACACACAGGAATGCTGATTGCTCTTTGTCACAAATAGATGTGGCAACAAAACTAG
CCACCCAATGGAAGGTTCAAAAGGCAGAAAAGCATTATCACAAAGCACTGGGCCACAGAACTTACCACTAAACAA
AGAAAAAAGCCACAAGCTGTGTTTAAAAAAACTTTAAAAAGTTTATAGAAAAGGCTTTTCTGTTTAACAAGACACC
TCAGTGACACTGGGCCCAGTAGCAGAGACACACAGGAAGCCTGCCCCACAGCATGCATCTCTCTCTCTTAGCTCCT
CCTGGGCTGTGCTGTTTTGTTTGGTGCTGCCACAGGCCCAGAAACTTGCAGTTGGACAACTGACATCAGCTCACGC
AGTGTACACTTTGTGATATTATTTTACTATACCTTTATAAAACGTTATTCTCTTTCTATAACACAGATCTATGAAGCTC
AGTAACTCCAGCTAAAAATCCTTACACAGTAATTTTGTAGTAAAAACATCTGAATGGAATCTATAAATTTTAAATAA
ATACGGTATAACAGATTGCCAAATGCCCTCCATTAGATCATAAAGCTACTAAGGATTTCTAATAAAAGTGTTTGTTC
TTGTTGTTTCTTAAGGAAGATAAAAGCATTGCTTGACATCATTGGAGAACAGATAAAAAATAGGAATGATTATACT
TGACACTTCTTTGGTAACAGGAGCCTAAAAGACATAATACTTAATACTGCTGGCTTCATCGGTATTTTTGTAAACCC
ATGAGCTCTGCTATGTTGTAACATAGAAGTGAAAACACCAATTCAAGTACGTTTTGCTAACAATCGAGGAAATGCT
ACACAGGCTTCACCACCCTGGCTGAGAGGCACAGCTTTGGCCACACAGCGCTTCTGCTGCCCCTTCTGGCCAGCTC
CTTGCTCCGTACACAACCGAAAGGGAAAAATACAATTCAGGTTCACGGCGCCGCCACAAGGCTGAACCCCGCCTG
CCCTCTGGGCGGGTCCGCCCGCGCGGCCATTCAGGCCTCCAGGGCCCTCTCAGCTCCCGGCCCCACGTCAGCAGC
CCGGGGCCGCTCCCCATGGGCAGCCCGGGCCGGGCCACTCCCACACAGCGCCATCAGTACGGGGATGGTGAGGA
CAACGCGGCGGCGGGGACGGCCGGACCCACCGCTACGTGGCCGCAGGGCGAGCCAGGCCCCGCACGGCCAAGG
CCGCGGAGAGGGGAGGCCGAGCCAGCAGCCACGGTGCCGAGGCGGGTCGGACAGGGGAAACGCAGCGGGGCC
AGGCGAGAACAGAGCGGGGCACGGAGCGGGGCACAGGCCTAATGCCGGAGCAGGGCGCCGAAGCGCCGGGAG
GCGGCCAGGCCGACAGGCTGCACGGGGCCCAGCGCCCCGCGGGCGCAGACCACGGCTCTGTCGGTCGGGGCAG
CCCACCCCGGCGGTGCACCTGCGCCGCGGGCAGCCGCTGTCCTCGTTGGTGTCCCAGGCCCGGCGGAGGCGGCG
ATAACGCTTTGCTCCAGCTCCGGCGCTGGCTGCGTTGAAGCGGTGGGGATGGGCCCAGGCTCAACGGCACTGAG
CAGTTAAAGGGCGGACGGTCGGCGTCGCTCGTCCGGCCGCGGGGCAGGCGGTGAAGGGCAGACTCGATGTTGA
CGCGCGGATTGTTCGGCCCCAGGCCAGGCGGCCCCAGGCTCTGGCCGCAGCTGCTCAATGGCGCCCGCCGCATCG
CCGCTCACCAGGCTCGCGCGCAGCACAGGGTCGCCCAGTACCGTCGCAGGAGCTATCGCGAGACACAGGGCCGG
CTCGGCTGCGGCTGCCGCCTCTCGCGAGACAGCGCGGCGGAAGCGCTCTCAAACTCTCGCGACGCTCTTTGAGGA
CCTTAGATATAGCCTCCTCTTTCTTGTTCACCGCGAAATCACTGCCACAGGAAGTCAAGGTGATGCAATGGAACTTA
AGTCAGCGCCGCCATTCCTGCTGCAGCTCCTTGTGGGGCCGGATGGGGTTGTTGTATCGTGTGTCCGTAATAAGCA
GGCAGGCCTTGAGGCTGCCTAGCCCAACATCGACACAGACTTGTGTGATCTCCTGTGGGGTGTCTCTTGTGCTGCA
CGACTTCCCTTGAGCTGTGGTGGCCCCCAGCCCCTGCTCAGTTGATCCTCCACAGGTATGGTGCCCTTTAACAGGCT
GCTCACTGCTCCTGCTGCAGGGCAGCTGGAGCAGGAGTATGTCTGAGTGGTGATTCAACAGGGCACGTGTGATGC
CAATGTGGTGGCTACGGGCTCTTTTGGGCGCTATGGTCTGCAGCATCACCAACGATTAGCCTACAGCGTGCTCCAG
AGTCACAGCTCTGCTGTTAAAGCTCCACCGTGAGGAAGGTCCTCCACAGTCCTTCATCATCTAGGGGCTTTAACTA
AAAAAAGAAATGGAAAGTGCTAACTCTGGGGGAACAAATAGGAAGTGGCTGAATAAGTGGACTCAGGACCAAG
GAAGGAGAAAAAGAACCGCCACAAAAGAAACCGAGCTCAGGCTGTTAAGGAACAGGGAATTTTAAGAGCCATAC
TGTGTTTGGTAGATCTTATCATCAAGCTTCAGAGAAGCTGTGAACAGTGATACAAAAGCTTCGGTGAATTTTTCAT
GGCTTTTTGAAATTGTCTTCCATAATCAAGCAAAATGCGCGTTATCAGCTAAGAAGAGTTCTTGGCATGATGTTATA
CAGTGTCAATAACATAAGGTACAGCACATATCAAGGTTGTGGAGGAGCAGAAAAGAAGAGACGGACATGGTTGC
TAGGAAACACTAGAGACTAATCACCACCAGGACCAATCTCAGAATGTATGTGGTGGTCAGGCAATGAGGTCTAGA
GAGGAGGTGAGTGACTCCACTACCCCTTCCAGGAAACACAACTCCATTACTCTCACCCGCAATATCACACAGCTGA
CCCAACACTTTCAGCTGATTAATCAGAGGTTTTGTGCAGTTTCCCATACAGATGCAGGCGTACACAGGTGCCTTGT
CCAGCCTGAGCATATTTTCTGTGGAAAGCCTTATGCATTTTTTGTTTGGGAAGCTCTTTAAGAAAACTGAAAAGTGT
TATTACTAAACATTTTGGTAGTGGTGAGGAGGAGATTGCTACAATCAAACTCTGCAGATAGAGGAGCAGTTGGAG
GAGGGAACCTTGCAAGCCAAGGTGTTTTGTCCTAGACCCATCTCCTTATCTTGTGAATACCTAGCACAGGCCTGTTT
AATTACAGAAAGCAAACATTGAGGAGAAAGAGGGTCATAAACTACACTTGCCTACATTTTGGTAGAGGACAGAAC
AGACCCATGAAGCCCTCAATTCCAGTATATTCTAAGTCTTGATTCTTTTGCTTGGGGAGGTAAGGTCATACCTTGGT
CGCGGTGATGTTGAAGGCTGATGACTATCTGAGACCTGGACTCCCAAACTTCTGGGTGTTCTGAAGAAGGACGGG
CACCTCTTAGGGGAGGAGGACATGAGCACTGCTCAAGGACGACTGCATCTCTGGGTTCCAGAATTGGTGACTGCA
TCTTTCTCAGAACAACCCCACCCCCTCCTTTCAAGTATGTTGTTTACCAGAAACAATATGTTCTGTGACCATCAGACA
TCAGTATCCCAAATATCTACAGGTTTAGTACAAAAACTCCATGCTTATGGGACTCTAATCCATCATATACTAAAGTC
CCCAGAATTGTAAACAACATGTTTTGTCTGACAGTAGCAAATGAAATTGCCTTAGAATATCATATAAACCAGAGGA

GGGAGCTTGTGAAATGTGGCAAGTACACAACATGTAACAAGTCCTGCTTAAAAATCCTGACTATGTCTGTTGGGCC
TGAAAGGCACGGGGAAGAAGCCTTACCTTGCCTAATAATGTTCTTTCTGTATGTAGGTGAAATGGATGTTGTTTTG
GGAGTGTTAGGGCTTTGACTTTTTACTGTAGGACAGAGCAAGCTAGGTTGTGTGTTGGGCAGGAGAGATTCAAAA
GCAGTCTGTTCGCTGTGTCATGGATATTCTGATGTAACGACTGACAGTGTCTGACTCTTCCCTTTGGCTAGGCTCTG
TCAGGTTTCCTTAGGTTTAGGCGACAGCAGCTGGGTGGTGTCACTGCTGAACATTCTGCAGGAAACTTCCCTGCTT
ACAGGATGCCTGTATCACTTTGGCCTTTCTATCATAGACCCAGGGATGCAAATGGGACTGTTGGTGACCTGTCTCT
TGGCCCAAGCAGCTGCTGGCTGCTATGAGTCTGCCCAAGCACCTTGAAGGGCAATGGTTAATGGAGAAAAGTATG
GAGGTGTCATGAGGATGACCCAGTTTGTCTCATGATAAAGCTAACCAATTCCACCTATACTCTTAATGGAGTGGTT
TCGTACTAAGCCAGTAGCCGGAGCGGCAGCTCAATGGCTGGATTTTGAGGTTTGGTTTTTTTCTCTGTGGGTCTGG
AACGGTTTATAGAAGATTTAAACATTGTCTTCTGAAACTCTATTTTGTTCCCACAGTGGATAAGAGTAACTGGCCCA
GCTGAGGGGGACTAAAGAACTCAGGCTCACGCTGCCTCAAATCAGTGAAGGCCCACCCAGGAGACGGAACGGAA
TTCAGTTGCGTTCTATTTACCTCGCGCTGCGGATGCTGAAACTGCTGCGGAGTTTTGACAGCGCAAAGTAGAGGCT
AGACGGAAGAGCGCTTCTGCGCCTGGAGTCAGGTGCAGACATAGGTGAAAACCAGGAATCTAACCGCTAGACCA
TGTGGGACTGCGTGCTCAAAGCCTCTGCTGCGCCGCCGGCTTCCTGCTCCGCCCGCGGTGCGGTGCAGCGCCGCC
TGCGGTCCGCGCTCGGTACTGCTGCGCCAACCGAGCGTCTGCTGCATTTGCCGGCGGGCGGACAGATGACGGAC
GGTGCAGTGAGCCGAGAGCTGTGGTGTGGTGGCGGGTAGTGTCATGAAAGAACAGCACAGAAAATCATACAGA
AACTCATTAAAAAAAACAATGAATAAGCCAAATGGAAGCGTGCTTCAGAGGAGCTCCTCTTTCACCAGAAACAAA
ACTATTTAATAGTATCAAATGGCTGCAAAGAAAGATACCCCGAAATGCTAAATTGAAGATAAAGTACAAAGCTGAT
GCAGATAGTGTCCATGTGCCAGCGAAGAAGATCCCAGCCAAGTTCCGGCCATCCCAGAATGGATATTTGCCATGT
GTGCACGTCGCACTGATGGCATTCCTCGCAGGATGGACTGCCTTGGCTTCCAGCACAGAGGCTGAGGCTGCAGCT
CGCTTTACTATATACTTTTGCTCAACGGTTGGAGCCTGTTCACCCAGGTGGGGCCTTGGCTGCAGCTGGTGGCATC
TGGTTCTGTGCATCAACCTGTCTGTCCTCAGCTTCAAGACCTGTCTGGGTGAACACATCTATGTTCTGGCTTCAAGG
GCTGCTCTTCACATGGGTGCCTCCATATTACACAAGTATCTCCTCTCTGGCTTGCATTATCCAGCAGCAGTTTTGGG
GTCTGTGCTGAGCCAGGACTGGGTTGTTCTGCTGAGGTGCTGCTTTAGAGTCTGTTGGGTTTCAGGAGCTCATGTC
CACCTCATGACCGTGGAAGGCCAGGCCTTGAGGAGCCAGGGCTGGACTGAGATCATCTGCTGAAGTAAGTGATG
TGCTGGCTTCGTGAGATCAGTAATGTGAAACTCAGAGAATTTTCATATCAGTGGGAAGCTTAAAGAACTAAGCTGT
CAGTTTTCTATATTTGCCTCCACAGACATGAAAGCAGACAGGTTGAAACTCAAACACATTTAATCTTTCTTTTCTCTA
ACTTTGCAATTCATGTCCCATCACAGTCCACCACACTGTGTGGCTTTAGGAACAGGTCTCATTGCACAGTCCCATGG
AAGTTGGTGGGTTTGCAGGCAGAGGTCTGCTGTCTTGTCTCAGCATTTCACCGCTGTCACTGCTGGGGGCAGTGG
TGGAGCAACAGGGCACTGGTCCCAGCACTGCATTTGGAGAGCTGTGGGCCACCCCAGCCCTGCACGTGCCCGAG
GCCACGGCAGCCTGAGCAGCTGAGGCTGAGACCGTAGCCATGGCAGGGGTGGGCTGGGGTGGGGTGGGCCTCC
AGGGATCTGTGGTTCTGTCAGAAGGCATTTGGGCTGGTAGCTCTTCTCCAGCTTCTGCACATATGTGCTACTTCCA
GCTGGAGTCTGTGAAAACTAGCAGCACCCCTTAGGCTAGACTGACCAGGAGGCTCTTGTCAAAAACTGGGTTGCT
AAGTCATCCTATATCACTGCACATCAGGAACAACTGAGAATGTTGCTATTACCAGTCCAGTAAGTGCAGCTCTGCT
GGCTGGGAAAGGTGATGTGTGTTGGGTCCAGCTCTCACTTGTGTGGAAAAATAGCCTTCATGCTGTGTAGACTGT
TCATCAATAAGCTAACACACCAGGCTGTTATCAGCCTGGCAGCATGCTGGATCCCAAGCACAGTGTGATGTGGACC
CCTGGCAGAAAAGTTAACTCCATCCCAGCCAGATCTAGTGCAGCACAAATACAGTATAAACTGCATGGGAGGTGA
AGACAGCTCTGGTTCAGCTGCTGTGCCAGCACCATACATCCCCAGCCACTGGAGTCACGCATACCATTCCCAGAAA
GGACTTCAATGCTGCTGTGTCACCAGTCAGGTTAGATGAGGGCACCTCAATGTTTGCAACTGCCTGACCTGGAAAG
GTGCCTGTCCCCATGTGTATAGCTCAGGAGGTCGCACTGGGCTTAGGCATGCATCAGGATGGACTTTGCAGTCTG
CACAGGCCCATAACCCATAGCAGTTATCTATCTACAGTTACAGCTGTAGCAGTATCACTAGTTATGCCCATCCGTGG
CTGCCCACTGCTCATGGAGATCTCTGTGGGTCTGACACAGTGAATGCAGAAAGGAAAGTCTGGTGCTGCAGCACT
GGCAACCTCTGGTTCAAACATAACTTCTCCTGTGGTTGCAAGTTGAACAGAGGATTGCTGCCATTTAATGGCTTCTA
CAGGACCATGGCTGGTCTTCAAGGGTCAAGTGCATGAAGGGTGAAGGATTCACCAAACATCAAGCCTTCTCCTGG
TATAAAAGTATCATTAGTATTAGTAAGCAGGCAGTGTGCAGGGCAGGTCTCAGGGCATCGTCTTGAATTAAAAAG
GGGATGCAGCTTTGTTAAAAATAAAATCGGTATTATTTCAACAAATAGATATGGTAAGAAAAGCAGCTAGAGATA
CTGCAGTTGAAGACATCATAAACAACAGCATGAACAAATCTTGGTGAAGGTGAAGAAGACGGCAGGAATCATGG
AAGATAAAACTGGAGACGTAATGTGTTCCCTGGACAGGAAGGTGGGTCTGGATGGTGAGCTCCCAGAGGCATCT
GGGCTGACAGAGCCTCTGCACTCATGGGCATGCTGCAGGAGAGCCTAAATCCCCTTCTTCATGTCTCCAGGTTTCT
GATGGGTGGGACTTTTGTCACAGAGGTAGAGTCAATTAATTTGTCAAGGCTTTGTGAAGAAAAAAAAACATTACC

AGTTCACAGGATTAGCTTCAACACTTTGAGTTCTTTCATCCTTTCTTGGCAACGAAATACCCCACAGTGTGCTACATC
AGCTGGCATGGCCATCACTTTAACACAGGCGGCTCAACAGCTTGTGTACAGGAGAAGGGAACAGCAGGCTGAAG
AGTGTGACAGAGGACATCTATGTCACACGCTCATGTTGCTCAGGTACAACATAGCATTCCCCTGCTAGTCTTGATCT
GCTTCTGTTGGCATCCCTGCCCCAGGCACCGTCCTGCAGCTGCAGTGGGACTCATGGCTAACCAGCTATGTCCCAG
TGCAGGACAGAGCACCACTGGCCTCATGTGTGCTGAGCTGATTAAACCTTTAGGGAGCTGGGCTGAAAGCAACAG
GGCTCTCACTGGGTACAAGGAGGTGTCAGGTGCGGAGTAGGGAGTCCTTTGGAAGCTCTAACTGACACAGTGGT
AGGTCACACATTTCAGTTCAGAAATATATCTGCTCTGTTCTCTCTGTGCTCCAGTCTTGCAGAGACCTAGGGTCTAG
GCAAGGCAGGGCACCTCTTCCAGTGGACTTATTCAGCCATCATTAGCAGAACATGAGAGTTTAGTTCATAAGTGAC
TTGAGAGCATAGCTTCCTTCATGTCTGCTTAGCTTTGGGACAAGGACTATCAGGGTCTGTGGAGTGTTTGGGTCAC
AGCCTGAACCTCTGATTGACCACCTGGTGAGCAGGGATTCAGCACGGGAGCACAGGTGAGATGATTCAGCTGTGT
GACTGGGAGATGGAGCCTGGCTGCACCTCTCCTAGAGCCCATTGGGGCTGACTGCCACTGGGGGTGGATCTCTTT
CTGGAGATCGCTCCTTGTGGATTTTCAGCACAGATCACAAACACCAGTGATTTTTTGCATTTGTCTCTGACTATCTTT
TCAATGTGATAATTTTGTTGCTTCTTCTCTATTTTCTATATAACCTGTATATGCTGATCATGCGTGAGACAGGATCGT
GAGACATTTCTTCTCTTTCCCTGCTTCCTTCATCCCTTTATGTACGGTTTGGGTTGTGAAACTATCTCCTCACCTGGCC
AGTCTGTTGACATGCAATAATCAGCCACATGCAAAACACCTATCACTTACCATCTGTTGATGGGCTGAGTGGTTCA
CTCATCTGGACTCTGGAATGAAGAGCAGCAGGAGGCAGGACTCAAACTTTGAGGAGTCCTCAGCTATCTCACTAG
CCACGACACCTTGTCCTGCAGGGCCTGTAATGTTAGTGCTATGTCCTTCACATATATCAGTTCACACTCAAAAGGCA
AACTATGATGTAGGGGGCGCTTGCATTAGACACCCTGGAAACTTTGCAGGCAGCCAGGGAGCCCACAGTAGCAG
GAAAGATGGCAGAAAGCCTCTGCTCTGTGTTTCAATCACAGTCTGTTTTAGAAAGCAAGACAAGAGCATTTGCTCT
TCCATCTGTTATCTATACATGCTACTAAGGTTTGAGTTGCAGCCAGCTGCTGCTATTGCCAATCGGTAACGTGATAG
TGTGACTGCTTACTTTGCAGGGAGCAAAGACAGTAGATGGAAAAAACAGGACAGGATGATTTTAAAAAGCCCCCA
TTGCAGAACACTGGCTTGTTATAAACATTGCACATCATTGCCCACAACTGAAAAAAGTGCTATTTGCTGTAGCGTTC
TTTTACAAATGGCCCAAATGTTTTCCACGTGGCTCTTCGTGTGATCTGAACCCTTGAGTCTTTCAGGCAGCTGCAAA
ACACAGACAGGGTTAATTAGAACAGGGGATGGTTTTTATTAGCTTCATACCTGCTAAGGCCATTTGTTGAAGAGGA
CTCTCATGGTGGATGCAAAGGAACCTCCAACCAGCAGCCCAGCATCGGAGGTCGGTCTGATTTTATTCAATAAAG
GATGAGACAATTTGCTGCAGGTGTCATGCTCCAAATAATGAGAAGAATTCTACATCTGCTTTCTTCAGCCTCAAACC
TCCTGACATGGAATAAGAGCCATGTTGGTCAGAGGCACCATTTTGGAGAATTGGCTAGCCCTAACCCTTGCTTAAG
GAGACCATCGCTGATAGCAATCTGGTCAGCTGTGGCTTTGTGCAGCTATGGAAACTCAAGGTTAGCTGTGTCCTG
GGCAGGCAATGCTTTGTCTTGATTACTGGTACAGCAAAGAACTGCCCATGGTAAAAATCTTTAGCAGGAGGACAA
TGTAGGTTGGAAAGACCTCAGGATCTTTACAGTCATCCCTGCCATGTTGTGACACATCCATCTGTGCCACAGCTACA
GACCTCGCCAGGTGCAACGCTTGAAGAGATAAGCAGTGGTGAGGTAACCAGATATTAGATGCTGTGGTCCTTTCA
TTATAAAACACACTGTGCATAGGACTCTTCAATACCAGAGACAGAAGGGTGAGAGGGAGGAGGGATAGGGGATA
GAGCTGCCACATTCTGAGAGATGAGGTGTGCAAAGCCAGTATCTGACCTGTGTGACTCTGCTCCCCCAGGTTGAG
CACTTTGTGTCCAGGCATTACACCTTCCTGGGGCCTGGTGGGAGCTGGCAGAGCAGGCTGCTCTGGTCCTGCTCCC
CATCCCAGCCCTGAGGGCTCTGCGTTGGGCTGGAGGCTGGGCAAAGCAAAGCAGTGTTACCTGAAGGTGCGGAT
CCCGACTGACACAGAGAGACAGGGGGCAGTTCTTACGTACGGTAACCAGCCATGCCAGTGGCTGGAGCCCTAGA
TACAGCCACCGCCAGTGGTCGGGGCAGCAAGAATATGCATTGCAGGTGGACATTTCCTGACGTCCATTTGAGGCT
TAGGACTAACAACAGAGCAACTTTCATGAAAAGAGAATGGCGTATGACCCTGGCACCGACAGCCTTCCGCCCACC
CGGGAACGGCTTGGCCTAGGAAGGAACCATTTCAGTGTGATCTCAGGCACCTGGCTCCTTTGGTTCTTCAGTGAAC
TAGTTGTGGAAATTGTCCTGCAATGGGGCTGGACAACAAGGGGATACTTTGGCGCAGGACAATTGCAGTGTCTCT
TAATTCATCTTTGCAGTGACTCGAATGTTCCACAGCTCCACAGTTCACAAAGGCTGTTCCTTCCAGTGCCTGATAGG
AAACATGGTTTCATTACTAGCAAAAGAGCTGGGGGTTTGTAACTGTGATGTCTTGCCACACTTTCAAAGACTGTTG
AGACATCTCGCGTCTACCTCCTCATGCTGACACCCAGGGTGAAGTAGTGGTGGCCAACCTGCATCATGCTGGCGTC
CTGTACCTGTCTGATACGACCTACACTGGAGGGAACCTCCTCAATACCCATTGCACCAGGCAGAAGTTTCCTCTCA
GCCCGTGAAGCCTGAGCTGACAGATCTGCAGGAGAGCCGCAGCAGGGCCCTGCAGAAGGGCATTTTCCCTCAGC
CGCCAGTCTTTCCCCCTCCAGGATCTGCCTCCCCAGGCTGCTGCAGCTCACTCCCACTGGTGCTCTGTGAAGCTACC
CTACAAAGTCAAACCCTGCCCTGGCAGCTCGGTGCCCTGTGCAAAACACAGCCTCACTGGGTCACTCTCCTGGTGT
TCCTGGGCGGATATGCTCTGCTCATCTCCCCTGTTTTGCAGTTTGTTACAGCGCACTGTTTGCAGTCTGTGTGAGGC
TCCTCTGGTGCAGTGTCAGGCCTGCTAACAGCACAAACAGCCTGTCTCTGCATGAACCACGACTGGAGGCTGTCAG
GAGGCAGTAAGCATTTCCCCAGATTTGCGTGCCCGCAGAGAACAGGTACAGCTATTGTGAAAGCAGACTTAGCAT

CACGTCTCTGCAGGAAAGCTGCCAGTTAACAACGCAGAGCTGTGGGATCCTTCATTTTGAGTGTGTGCTCCCACTA
TCCGCCTCTGCACTGCTCCATGCCCCAGCCCTCAGTTTTGCTGTCTCTTATCCTCCTGTACTCGCCCCATGAATGCCA
CCAGGGATGTCCTGGTGCTGCTGTTACCAGGGCCCACGCTCCATCTGTGCTGGATCCATGCGAGCAGGCAGCAGT
GATCTCACTGTCTCTCTGTGTTATTTACAGTGGGGGGACTTTGCTCAAATATTCTTGGGTTAAGTTACTCACAGGTT
CAGCAGTGCAAAGCAACATCAGCGGCCTTAACTGCCCAAGGGCTGGTGTACAATTATTACTGATGGGCTGAGGAG
GGTCATGTCACAGCTGTGCTCTGTGTGAGCCTGCAGACCCCCTGCTGAAGAGCAAGAGCCCACCTGGTGTCTGCC
ATCTCCTGCCATGGGCAGAGCCTCTGGGCTCGGGCTTTCCCTAAGCTGCTCTGTGTTGTTCCTCCCTGGGACGAGA
CTTGCTGTCGGCTCTGCTGTGCCGCTGGCGATGTTGCTCCATTAGCGGAGCTGTGAAGGCTGTGCTGACCAGGCA
GGGACTCAGCTCTTTACTGCAGCGCTCCTGCTGCTGAGTTGTCAACGCTCTGCCATGCCCATGAGTGGTGCTGTTG
TCCAGTATGCGATGGTTGGCACGGTGCTTGATGCGGCGCTGGGTGTTTTGCACCTGCGCGCTGCCGGGCTGCCGG
CGGTGGAGCACCGTCTCCATCGAACAGCTGCGATGGCTGGCCCCAGGGGCACAGCCATGGCACAACGCTGCTGCC
TGCGCTCGCCCGGCGCCCGTGTCCCTATAGATCATTTCGGGGACACCGCACCGCTCCAGCCTCCCCATCGCTCAAA
TGCTTCTACCAGGTACCCCGGCTCCCCTGGGTCCCGTTGTCCCTCAACAGCCCTTCCCATACATGTCTGGTCATCTC
GCACCCGCCCGCACCGCCGGCGAGGTGGACGCGCGGACCGCAGCCGTCCTCCGCTCCCTCGCTCCCGAATCGCCG
CTGCCGCGGGAACGCCCTCGTCCCGCGGGTCTCACGGCCGCCTCCGGTCCCAGACTGCGCCGAGCTTGCCGCCCC
GCCCCCGCCGCTCGGCCCCGGCCCAGCCGGCGCGGCGCTGTGCCAGCGGCCCCGGCAGAGGACCCGAGGGAGCC
CAGCCAGGGTCCCCAGCGGCAGCGCCAGTTGGCGAGGCAAAGAGAACGGGCAGGGCGGCGAATCGAAATTACA
TCCCTGCCCGCTGCGGAGGCGGTGGTTTGGCAGAGGCGGTTATGGCGGAGAGGTGAAGGTGCAGCGCCACCTGT
TGAGGGCTGCAGGCCGCGGGCAGAGCCCGGCGGGGCGGGCCACGAACCGCGGGCAGGGCGGCCCGGAGCCCC
CGTCACGCGCGGTGGGAGTTGGATGGTGCGGACTGCAGCGCGCTCGGGGTCCCCGCGGGTAAGGAGCAGGTTG
CGGGTGCTGAGCGGCCGTGCCGTGCAGGGCTCCCGCGGGGCTGGGTGCGGCTCAGGCGGGTTTCACCCCAGCGA
GAGTTGCTGTTGCGCTCTTCTCCGGAGGCTGCGGCCGGGACACAGGGAGTTCCACGGGCGTTCGGTGCCGCTCCC
TGTCCCCCCGCGGTGGTTTCGCAGCTGCTCGAATCGCCGTCGGAGTTGATCCGCAGCGCAGGGTCGGCCTGTGTT
CCGCAGGCCGTGCCGGTGCCGCCGCTGTTCTGTGTTCTGCTTTCCGACGGCCAGAGGAACCAGGCAGTCGGACGT
TCCTGCGCGCTCCGCGCTCCAGGTATGGAGCTGGCCTCTCTCCTCTTGTCTGGGGGAGAGCCGTGCTGGCGCCTTG
CCCGGCGTCTCAGTGCAGGCGCTAGAACTATCCACTGCTGCTCGGAGCGTGGAGTTTGTCTGCCCAGCTCTCATCT
CCTCCGCAGCACGGTCCCAGTTAGTCTGCAGTAGAGGCGTCTGCGTTGGAAGGGAGCTGGACCAGTTCACCGGAG
CCCTTGTTTAGCTCGGGGTATTACGTCTGCCCACAGCCATCATCACCGCTGCTTCGGCCTGCGCTGCACAGCAGCG
ATGGGACTCGAAGCACTTGGGAGCAGTTTGCAGAGCTACAACTCTGGACCAACCGGCTCTACTCCTTGTGCTGCTC
CCGTCCTGTCCCCACTGCTCTTGATTGTTCCCTGGAATTGTGTTGTTTGCGGTCGATATGCTCTTTCACTGTGGGCA
GTCATCAGAAGCTCTTTGGAGGCCGTGCTGAGAGTACGCGGTGGTTGTAGAGATGCTGGATTTTGCAGCCAGGTT
TTTGGTGGGATGGTTACATGCCTTTGGTATTCACAACAGGTTTGCTTAGCAGTAGATGTCTTGGCCTTGCTTAGCTG
GAAGCACATGGACATGGAATCCAAGTGTGCTGGCCTGGTGCATTTTGCAACAAGAAAATTTATAGCCCTCCTTGTG
ATGCAAGGAGCTGCTCCAGCCTGACAGTTTACTGGATCTGCAGCTGAAAACCTTGCATAAGACTTGTGTATCTACT
GTATAACCAAGTCACGGAATCTGGTTCACTCGATGCTGCCTACTTTAGGGGCAGATGTCCTACTCAGCATATGTAC
CTGATCTCAAAACGTGTGCACTGCTGGTAGGGTGGAGGAAGGGGGAGGCTGAGGCCGCTGCTGGGCAGCTGGC
TCAGTGTGTAAAGAACCTTGCAGGCATCTGTGTATTTATTTAAGCTACAATTTCTTTTATCAGTCAATCACTGTTCCT
CTGGGGCTGGCTTTATAGGACTGGGGCAGCTGCCTGCTTTGTTGCAAACAGGTGAAGTCTCGTGTGCCAGAAACA
GTTGCCCAATAAAGCTGAGCCAGAAGGATCCGGCAGCACCGCTGGGAGGCTGGGAAGTAGGATAATAGATCCAA
AATTGGCTCTGATGCGAATTGCTTCTGTAATGAAAGATAGGGAGCATCAGCTGCTGTAGGATTCTGGCTGTTTTTT
GACCCGTAGGGCTTCCTTTTCCTCCTTTTCCCCCAGCAGTACGGCTTAATGAATATTCATAGGGTTCCTTTGAAGTT
GCAAAGATGAGTCATATCTGTATTTCCTGATGAGCAAATGCGCTGGTAAGCCTGTGGTTCGGTTGCCATTTGCCCT
TGAGAAGCTGCTCAGCTCGGTGCACGGCAGCAGTTTGGCTATTTGGAACCAAGGAGCCCCATAGGCTTTGCAGAG
GAGATGGTGCAGGCGCTGGGCTGCAGCCCGGCTGGTGAGGCTGGGATTGCCTGGGTGCTATGAAGGGCACTTGT
TTGGTGAAACCTGCAGCTCTGGCTGTGTTTTGTAGCTGATTAATGCAAAATAAAAGGATTCCTGCGCTTTATGTTCT
GATTACAAACCATTTTGAAAGTGAGTGGTTTGCTCTTGATCTCCAGTATCCTGAGTATTGAGTTTGTTGCAGCTAAT
TAATTCAGGGTTTAATGTATGCTTTCGAATACCAGAACTAGACTAGCTTGTGTGGGGATTTTTTTTTTTTGCTCTTGT
TGTTTTTAGAAACTGGCTTTAATTTAGCAGTAAGTGAAAGCACCCGCAGCTGGCGCCTCCCCAGCGCCGTCTTGGC
TCCTGCAACAGCCGCTCGCCCAGCAAGCTTTGCCGAGTCGAGGCTCTTCTGGTTTTGGCAGCATCTGAGACCTCCT
GCTGGTGAACTGGCCCGGGTCCCCGCGCACAACCTCTGACGGAGGCTCTGAGAGCCCTGTTGTGCGCTCTGCTCTT

GTTTTAGCAGTCTCTTACATAGGTCCCTGAATAGAGTAAATGTCCCGCTCTGTAGTGAGCCACAACTGCAGGAGAA
GCTTCTGTTCCCAGCCAGCATCACAGCTCGTCTGCTGGGTGAGCTATTCCTGGCTGAGCACCCGGTCCTGCATTAA
GTAGACAGGTTGGGTCTGCTTTCTTGAATCAGCACAGGTGTCAGAGGTTATCCAGCAGGATAGCCCTGCCCCCAT
GGAGGGTGCTGCTGGGCAGGTGTGGTAGGAGTGCTGGTGTGATGTATTGGGATGTAGTGGTTTAGGACAGAAA
AGACAGGTGGGTAGGAGGTGGAAACTGAAGGAGGTAGCATACAGGGACCACCAATATGCTGGATGGAGGGATA
TTTATAATACAGATAGTTATGTGTATCATCTGAACAATAACTTAAACAATATTTATAGGGATATAACAACAGCGTTG
GGTGGATCTCTGCCTGTTGCTCTGGTGTGTGACTATGCAGATAACCTGGTCTTTAGAAAACACCTTCCAGAGCTGC
CACCAGACTCTTCTGATGGTAATCAAGGACACAAAAGGAAAAGCTATTTTCTTCCTTCTCTGTGAATTCCTTTCCCA
GCTACATTCCTGTTGTACTACCCAAGTGGCTTCCTTGGTGGATGGTGGTGGAATGCTAAAGAAAAGCTTTGATCAA
GTAAAGCACATGAATTTTGAAGCGTTCCATTTGACAGTCAGTCCTTGCAGATGAAACAATAATTTGTGCCTTGTTGT
GACATGTCTCTTTCAAAACCTCCAGGCAGATCTGAGTAGTTATTGAAGCACCCTCTTGTTTGTAATAGAAATAAATT
CCAGAGCAGACATTGGGATCTGGTTTAGCTGGATTCCTAGGGTTCCAAGCTCTGCTGTCATGTTATGTTTGATGTT
AATTCCTCTCATCAGGTAGCAGAGAGGCAGTTCACTTATTGGGAAGAGTGGCTGTAATGATCAAGTAACTTACTGT
ACCTCTTCCAGAGCTCAGCCATGAGTGTGCTAAAGCAAACACTCTGATTTATTTTTCACATGTTTCATGAGGGGAAG
GTAATTTCTGCTTTGTGCAACTTCTTAAAAAGCAATGCTTTATCCTTCTCTAGAAACCAACTGAAGCAGCAGACGTG
GGGTCCATCTGGCTCACGGAATTAGGCACTCTAGTCAAAGAGATTTTCTGTAGTTCTTAGTGTTTTCCTGTGATCAC
GGTTTTCATCGTGAGAAAGTCTAAAAAAATGTGATTTATTTGGGATATATTCTCTTCAAAGTACTGTTCATGTACTT
GGAGTTTACTGATGTGAACGCATTGTTTTTATATTTCCAGTTGAATACG

>10.319. 6224383f-03fa-4abb-ba54-84ea90056737 protocol_group_id=2020-02-09_1st ch=291
barcode=BC02 read=9949 start_time=2020-02-09T16:36:45Z flow_cell_id=FAK79217
runid=88172aec3f27372c4684111da770399a9fd1fb40 sample_id=2020-02-09_1st

TGGCGTGTGATTTCTGCCCAGTGCTCTGAATGCTCAAAGTGAAGGAAATTCAATGAGCGCGGGTAAACGAGGCGG
GAGTAACTATGAAACTCTCTTAAAATTGTTTAATAAATGCCTCTCGTCATCTAATTAGTGACAGCATGAACAGGATA
GCCTGAGATTCCTGTCCCTACCTACCGTCCAGCGAAACCACCCCAGCCAAGGGAGCGAGGCTTGGCGGAATCAGC
GAGAAAGACCCTGTTGAGCTTGGCTCTGGTCTGGCGCTGTGAAGAGACATGAGAGGTGTAGGATAAGTGGGAGG
CCCGCCTCAGCCCAGCGTCATGCGGAGTCACCCACCCGCGCATGAACCGGCGCGGTGAATGCCACCACTCTGATC
GTTTTCACTTACCCGGATAGTGACCTTTGAGGGTCTCGCTTCTGGCGCCAGCCCGGCCGCTCAGCGCGCCGGTAGC
CGACCCATCCGAGGACAGCGTCAGGTAGGAGACGGCTGGGGCGTACACCTGTCAAGCGTAACGCAGAGGTACAA
ACAGCTCTAAGGCGAGCTCAGGAATAAGAAACCTCCCCGTGGAGCAGAAGGGCAAAAGCTACGCAGTCTTGATTT
CGAGAATGCAGTGCGGGCCGTGAAAGCGGGGCCTCACGATCCCTTCAAAGGCTCACCGGGTTTGCAGGCAGGAG
GTGTCAGAAAAAAGTGCAGGGATAACTGGCTTGTGGCGACAGCGTTCATAGCGGCGTCGCTTTTTGATCCTTGAT
GTCGAAACACTTCCCCTATCATTGTGAAGCAAGGAATTCACCAAGCGTTGGATTGTTCACCCACTAATAAGGAACG
TGGGCTGGGGTTTAGACCGTGGGGATTGGAGTTAGTTTTACCCCCTACTGATGATGTGTCGTTGCGCTATTAATCC
TGCTCAGTACGAGAGAACCGCAGGATCAGACATTTAGTGCGTGTGTGACGAGGAGCCACTGGAGCGAGGCTACC
ATCTGTGGGATTATGACTGGAGGCAGCCTCTGGTGAATCCGCCTAAGCGGACGATACCGCAGCGCCGGGGGCGC
CTCGGTGGGCTCGCGATAGCCGGCCGGCTTCCTTCGCTGGCAGTCGGCGCCCGTCAATGCGTCAGAGGGCGGGCT
TGCGGCGGAAGCGGCGCCGCCTCTCACCATGCGTCCTGCTGATGATAGAGGCCGGTGCTAAATCATTACCGTGGC
TGACCTGATTCTGGGTGGGGTTTCGTGCGTGCAGCAGGCAGCTCCCTGGCTGCGATCTGATCAGCCCTCGACACA
GCTTTGTGCGGCCGGCTCCCTGAGCAGGCGAGCCGAGCAGGAGAAGCGCTGGCTGCTGCGCGGCGACAGCCGG
CCAGCGCTGCCCAGCACAAAGCACCCGCCCCCGGTCTCGGGTCACCATTCTAGTTCTACCGGCTTTGCGGAGGTGG
CGCGTCTGGGTCAGACCCTCAGTGTCTTGGCGCTGGCGGGCCGGGCTGGGAGACCCGGCTAGCTGCTGGGTTGC
TTCAGTCACTCTGTTCAGGAGGGTTCCGTGCAGCGCCCGGCGCAGTGGAAAGAAGGGGAGGTGCGGCTTGGTCG
CGCGGGCGGCGTGCCAGTTGCCCAAGCCGATTTTCCCCCTCCATCCACCCTTCCGGCAGTAGGCGGCCTTCGGGGT
GAACCTGAAGTGACCATCCGCTGTCGGCGGGTGCAGTTGCGTTCCGTCCGTTACGCGGGGTAGGCGTGTCGACCG
TAGCGAGGTTGACCTGTGGCGGTGCCGTTTTTCCGTCCGTACCGCCCGGGTAGTGTCAAGCAGCAACGCGCGTGC
GCAGCGCTGTGTCAAGCGGTAAAGTGCGGCGAGCGGCCAGCCCGACCATTGACCCGACTGACCCGACCCGACCC
GACCCGACCGCTTCCGCGTTATTGCTATCTTTTTATTATTTATTTTATTTTATTTACACGCGACATTTCACGATCTATC

TGTACTTTTTCACAACTATCTACTTCTACTTTTCTTTGACTTCTGCCTTCCCTTCCCTTCCTTCCCTTCCTTCTTCCCTTC
CCTTCCCTTCCTTCCTTCCCTTCCCTTCCCTTCCCTTATACTTCCCTTCCCTTCCCTTCCCTTCCCTTCCTTCCTTCTCCTC
CCTTCCTTCCCTTCCTTCCCCTTCCCTTCTCTTTCTCCTCGTGCCCTTGGCGTGTGCTGTTGGCCAAAACCTCTGTCAG
AAGAAGGGGGTCGCTGCGTGGCCGGGAGGGGATGGGGGTCCTTGACGGAGTTGCGGTATGCGGTAGACCTGGC
TGCACCCGGCGGCGCCACCGATCTTCGGCCCCGCGTCCGTCTTCCACCTTCCCCACCCCCGCAGTCCACCGCCTGGC
AGGCCGATCCAGACAGCACCCGGCGGCGGCCGCCTCCGCCTGCCCCACCGGCCCTCGCCCACAACCGCCCTCTCCC
GGACATCTCGCCCGACCGGCCCGCGACCGTGTTTTTATTTGTTGTTTGTCTTTGTTTTTGTTTTTTTTGTTTCTTTGGT
GATGGTCACTACTTCGGTTATTTGTTTGGACCCGATTGGCCCGTTTCAGTTTTCTTTTCTTTCCCCCTTTTCCCGATTG
CTCAGCGGCTCGGCCCAATGGCGTGAACTCAAACTTCGGCGACCGGAGCCATAAACACTCGCCCAACCCCACCAC
CCCGCCCCTCACTCTCCCGGCGAAAATCCCATTCAGTTCATGTTTCTCTGTCACCGTGGGAGGGGCTGGCTGAGCG
AGGGCGCGGCCGCCTCCCGCCAGCCCACCTCCGCCGCTGCCGTCTTCCCGCCGCTCTTTCAGCCATCCCTCGCCACC
GCGGCCTGCCCATGAATGGGATATCCGGCCCGACGGACCTTGGTGGGCGGGAGCCGCGCACCAGCCACGCTCTT
CCTGCAGAGCCGGCCCCAGGCGGGGCGAGCGGCATAAGTCCCAAAAAGGGAAAGCAAAGAAAGAACCCACAAA
GGAAAAACAAACCAAAGCAAACAAGCAGGAAAATAATAAAACCGAGGAGGCGCGTGGGGGCACGGACAAGCTG
TCAGACGGACAAGGCTGCGAATGACGGACGAACAAACAAACACGCAGACAAAGGCAAAACCTTGCCCGGTGGCG
GTGGCGGGGTGGCCAGCTTCAAGCAGGGGCCGGGCCGAGAAACCAGTGCGCCCGGCTTCGGCCAGTAGCGGCC
GGCTCAGCCAGTGCACTGGTGGGTTCAGCGGCTGCGGCCGAAGTGCGTCTCCGTGGCGCCTCCGGTGCTCCGGCC
TGGTCTACCCGGCTCCCGAGCGGGCCGTGGTGCTTTCGGTGGCGGCTGCTGGAAAGGTCGCTGCCTATGCGGCTC
GACTAGGTCGGCACGGTCTGCCCGGCTCCGAGAGGAGCCGTGGTGCTCGGCGGCTGCTAGAGTGGCACCCTGTG
CGGCTGACTAGGTGGGGCACGGTCTACCCCTGCTCCGTGCGTGCGCGAGGGCTCGGCGGCGATCGCCCAGAGTG
TCCTCACCGTGCCAGCCCGTCCGTCAGCGACCTCCTGGCGGTCATGGCCCAGGCAGAACAAGGCTGTGCCCGGCG
TGGCTTTCCGGCCGCGCTCAGCGACCCCTGGCGCCGCCGCGCTGGGCCGGTCGCTCGGGCCGGGTAGGCCGTGC
CGACCAACTCGAGCCGGACGTGCAGCGACCGCTGGCCTTGAGGAGGCTGCGTGTGCGCCCGGCACGTTCAGCCT
CTGGCCGGGCTCCGTTCCCGGATTCGGGCGTTCCTCGGTGAGGTGTCGGGTGCTCAGGCAGCGACTCGAGTGCG
GCACCCACCTTCACGGGCTCGCTCGGCACGGACTTTCCCTCAGTGGGCGTCGGCGATCTGAGGCCGGCGGCTCAG
TGCGCCGGCCGCGGTCCAACGCTCCCGGCGGTTTCCGCTCGGCTCGGACGCTCCTGGATGGCGGGCGATCAGGC
GCGGCGGCTCAGAGTGCACGGCACGGTCCAACCGCTCGGCGGGCTCGCTCGGCTTTCGGAAGTTCCGCGACGGC
GTCAGGGTCTCAGCCGCGGAGAGGCTGGGTCGCCCGACACGATCCAGCCTCTCCCGGCGGGGCTCCGCATTTCGG
CTCGGGCGTTCCTCGGCCGGCGTCCGAGCGATCTGAGCCGCCGGCTCAGTGCCTCAGCACGGTGGTTGACCGCTC
CAGCGGTTCAGCTCAGCTCGGGCGCTCCCTCGGATCGGGCCCCCGTCTGGCTCGCGGCGGCAGGTCCCAGCCTCT
CAGGCGGACTCAGCTCGCTCGGGCGTTCGCGGAGTGGCCAAGTACCGCTCTTGTGGCTGCTCACTGGCACTGTAT
TACCCGGCTCCGAGCGTCTCCTGAGGCGAGGGGGAAGGGAGACGCGGCCGGCCGGCGCGGCTGCCCATGCGGC
TCGACTAGGTCGTACGTTCTGGCTCCGAGCGGGCGTGATGCTCGGCGGCGGCGGGTAGGGAGTCGCTGCCTATCT
TGGCTCTCGACTAGGGTGGCGCGTTCACCCGGCTCCCGGCAGGCAGTGGGGTGCTCGAGCGGTGGGGAGTCGCT
GCCTGTGCGGCTCGTCTGGGTCGGCCACCCCGGTCTGCTGCCGGCTCGAGGCGGGCCGTGGTGCTCGCCCGGCG
GCGGGTAGGGTCGCTGCCTGTCTCACGGCTCGACTAGTGGGCAGTCTGCACCCGGCTCGAGCGAGGCGTGGTGC
TCGGCGGCTGGCGGGTGGGAAGCAAGCTGCCTGTGCGACAGTCTGGAGTGGCCCTGGTCTACCCAGGCTCTGGC
GGGCCAGTGGTGCTACGCCAGGCGGCGAGGTAGGGGCGCTGCCTATGCGGCTCGACTAGGTCGGCCGCAGGTCT
GCCCGGCTCCCCGAGCGGGCCGTGGTGCTCGGCGGCGGCGGAGTAGGGATTAAGCTGCCCTATGCCAGGCTCGT
CTAGGTCGGCACGGTCTGCCCGGCTCCCGAGCAGGCCCGTGGTGCTCGGCACGGCCCGGCTGCTAGAATTCGCTG
GCCTATGCGGCTCGACTGAGTCGGCAGGTCTACCGAGCTCAGGCCGGGTGGTGCTCGCGGCGGCGGCTAAGTCG
CTACCCGTCGCGGCTTGACGCCGCTGCCAGGTCTACCCGCTTTCGGCTGACTTTGCCGCCCTGTGAGATAGAGCGC
TAAGCTCCGGAACTGCTAGAGTCGCTGTTGTTTTCTTACCTACCGTTAAATACATTGTGAGGCATTCTCGGCACCGC
TGCCATTAATCGGCGCGTTCTTTTCAGCCAGTTTTTTCTTGCGCCTTCCGTTTTCGTCTGGGAGTGAGCGCGTGCTG
CGTCTCAGAAGCGGCCGCGGCTGGCGCAAGCCCCAGCAACGGTTAATGCTGGGCTGCGGAACGCTTTAGGTTCA
GCTTTCTAGTGCGCCGCTTGTTCCCTTCGGTCGGTGGCCTCCGTCGGTCGGATGTTTCGCTGCCCGTTGTCGCAGTT
CAAGCCCCGGACTCTGGCGTTCGCTTCGTTCCGCGGCGGAACCCTCAAATCGGGCGAAAAGCGAGAGGGCGTGG
GCAGGCGTCTCCCCGCGCCGCTACCCATGGGAAGAGCCCCGAGAGGGAGGAAGGAAGGCAGACAGACAGGCAG
AGGGAGAGAAAGGCGCAGGCCCGGCGTGCGGGCGGCCGGCAGTCGGACCTTAGAGGGTCGGGCGTCCCGCGG
GTCCCGCCGTCACGAGAGGAGAAGGACCCACGAGGGCTCGGCAGCCGCGCGGGCGCCCGCGCGTTCTCCCTTTC

GCACCGCTCTACTCCTACTTGCGGAGCCCCGACCCGATCCACCGTGGCGCTCCTGGGGCGCGTCGGAGGAGGCTT
CCCGGCGGAGCCGGCGCAATCCCGCTCCAGTATGCAAATTCGGGCGGCGTAAATGAGCAGTGTTCAGGCAGAGC
GAAGGCACCTCTCGGCGGACGTTACACACCCACACGCACGTGCGCGTCGCGGTCTCACACCCCGCGCCCGGGTGC
GAAGGGCTTCGTGCCTTCCCCACCCGTCCTCCTCTACACAGCACCCTTACCCCTCCCGTCCCTCCTGCTCCCCCCTTC
TTTCCCGTCCGATCGATGGGGCCATTCGGGTCGCGTCGGTGGCCCCCGGCAGGCCTCCGGCGCCTCTGCGTTCCCC
GTCCCGGGGAAATCGCTGCGGCGTCCGGTGTTACAGGCTGCAGGCGGTCTCCTCTCCGGTTCGCTTCCCGTCGTGC
GAGTGAAGCTCCGGCTCTCGAGGCGGCCGAGGAAACCATGACCGAACCCCGCAGTGTGATGGCGGCCAGAGCG
GCCGCAGGCCTGGGGTACCCAGTGAGTCGTGAGCGAGCCGGCGCCGACCTGTTCGGGGACACGCGTCTGCGGCA
GCAGAGCGACGGGGTGTCAAGCATGAGGAAACCGTCCCGTTCGGGAAGCAGGCAGAGGCTAAGTTAAGGGCCA
GGGCGTCCGCCGCCTCCCCTCAGCCCTCCCGGGCCGCCCGGATCCGTGCGTGGGGTTTCGCCTCGACGTTCCGCCC
GCTGCGGGCGTGCCCCGCCTCAGGAAAGGAGGTCTCCCGGTGTTGGGCGCGTCATGCCGAGGTCGCCTTCTCCTC
TAAGCGCGTCCGCCAGTCTCGGGCGGCGGGGTTCGTTCCCACGGCCCGCGGTCACGGTGGTCAGCTGACGATGCC
GCCGTGCCGTGCCGTGCGCTGCCCGGGCTCCCGCGGCGTGGCCCAGGCGCGCCGCCGGCGAAAGGCACGACGAC
ACTTACGAGCTGCTGTGGCGCCCGCTCCTTCCCGCGTGGGCGCAGTCCGCCTTCGGGGACGAGGCGCGCCTCTCC
GCCGCCGGTCGCCAGCGGCGGCGTGTGGCGGCGGAGGCACGCCCCTCCCGCCGAAGGCGTTCGCCCCGTCGGCC
ACCGCCGTGGAAACCCTTGAGAGGAAGGGCGTTAGAAGC
