# Supplementary material for: ﻿Description of the complete rDNA repeat unit structure of Coturnixjaponica Temminck et Schlegel, 1849 (Aves)
Source: Comp Cytogenet. 2024 Sep 25;18:183–98. doi: 10.3897/compcytogen.18.127373 (PMC11447458; doi:10.3897/compcytogen.18.127373)
Supplement: Supplementary material 2 — Localization of the promoter and transcription start site using RNA-seq data mapping [file comparative_cytogenetics-18-183_article-127373__-s002.pdf]

Localization of the promoter and transcription start site using RNA-seq data mapping

Confirmation of the theoretically predicted position of the RNA polymerase I promoter within the described intergenic spacer of the rDNA repeat unit and determination of the transcription start site location was performed based on transcriptome data mapping from NCBI SRX9608520, SRX9608583 and SRX574377.

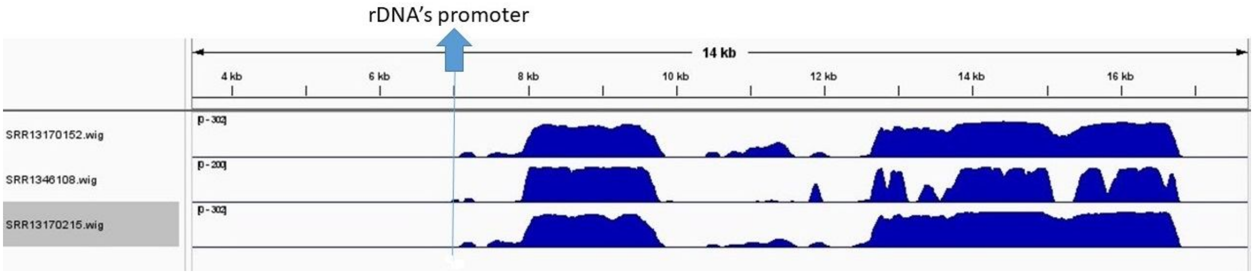

RNA-seq data were downloaded using SRA-toolkit (http://www.sthda.com). The reads were trimmed using the TRIM_GALORE program (https://www.bioinformatics.babraham.ac.uk). Mapping reads were conducted using BOWTIE2 (http://bowtie-bio.sourceforge.net). The resulting SAM file was converted, into a BAM file sorted from duplicated rows using SAMTOOLS (http://samtools.sourceforge.net). Then we converted BAM file to WIN format using the following script: https://github.com/MikeAxtell/bam2wig. The sequence coverage was calculated using SAMTOOLS. We visualized the coverage and found the starting point of transcription using IGV browser (https://software.broadinstitute.org).
