# Supplementary material for: ﻿Description of the complete rDNA repeat unit structure of Coturnixjaponica Temminck et Schlegel, 1849 (Aves)
Source: Comp Cytogenet. 2024 Sep 25;18:183–98. doi: 10.3897/compcytogen.18.127373 (PMC11447458; doi:10.3897/compcytogen.18.127373)
Supplement: Supplementary material 3 — Composition of tandem repeats in the noncoding spacer sequences of Japanese quail rDNA repeat units [file comparative_cytogenetics-18-183_article-127373__-s003.pdf]

Table S1

Characteristics of ITS1 tandem repeats

№	Repeat	Inverted repeat	Structural feature
1	$(CCG)_3$	$(GGC)_3$	-
2	$(CGGG)_3$	-	-
3	$(GCC)_3$	-	-
4	$(GCG)_3$	$(CGC)_3$	-
5	$(GAG)_3$	-	bendable DNA
6	$(CCT)_3$	-	bendable DNA
7	$(TC)_5$	-	bendable DNA

Table S2

Characteristics of ITS2 tandem repeats

№	Repeat	Inverted repeat	Structural feature
1	$(GCC)_3$	$(CGG)_3$	-
2	$(CGA)_3$	-	-
3	$(GTTC)_4$	-	-
4	$(CG)_6$, $(CG)_5$	-	-

Table S3

Characteristics of IGS tandem repeats

№	Repeat	Inverted repeat	Structural feature
1	$(ACCCG)_5$	-	-
2	$(CCCGA)_3$	-	-
3	$(CCGAC)_3$	-	-
4	$(TTTTA)_5$	-	-
5	$(CTTCT)_{18}$, $(CTTCT)_3$	-	bendable DNA
6	$(TTTG)_3$	$(AAAC)_4$	-
7	$(CCT)_3$	-	bendable DNA
8	$(AAT)_3$	-	-
9	$(AACA)_3$	-	-
10	$(CGG)_3$, $(CGG)_4$	$(GCC)_3$	-
11	$(GCG)_3$	-	-
12	$(GGCTC)_3$	-	-
13	$(CG)_5$	-	-
14	$(GACCTGACCC)_3$	-	-
15	$(GACCT)_4$	-	-
16	$(TCT)_4$, $(TCT)_3$	-	bendable DNA
17	$(TCTTC)_3$	-	bendable DNA
18	$(TC)_5$	-	bendable DNA
19	$(T)_9$	-	bendable DNA
20	$(GAGGGG)_3$	-	bendable DNA
